# A Magnetic‐Driven Self‐Rotation‐Stabilized Miniature Swimming Robot

**DOI:** 10.1002/advs.77382

**Published:** 2026-09-09

**Authors:** Fujun Wang, Chuhan Zhang, Hao Zhang, Yanling Tian, Cunman Liang

**Affiliations:** ^1^ Key Laboratory of Mechanism Theory and Equipment Design of Ministry of Education Tianjin University Tianjin People's Republic of China; ^2^ School of Engineering University of Warwick Coventry UK

**Keywords:** magnetic‐driven, miniature swimming robots, polyhedral deployable structure, resonance‐driven strategy, self‐rotation‐stabilized, water environment monitoring

## Abstract

Miniature swimming robots (MSRs) are crucial in fields like environmental science, industry, and healthcare, especially in accessing narrow underwater spaces. However, current disturbances and undercurrents in real waters can severely disrupt the trajectory and stability of the MSRs, preventing them from effectively completing their intended movements and maneuvers. To address this challenge, this paper proposes a magnetic‐driven self‐rotation‐stabilized miniature swimming robot (MSMSR), inspired by the *Dipterocarpus alatus* seed rotation mechanism. The robot features a polyhedral deployable structure (PDS) with flexible joints and four inclined magnetized feet. These flexible joints enable energy accumulation for rapid swimming, while the PDS facilitates pulsating propulsion. The magnetized feet support rotational movement, allowing stable self‐rotation swimming. Additionally, a resonance‐driven strategy, based on the coupling of the robot's natural frequency with the magnetic field frequency, enhances its swimming performance. Experimental results show a maximum swimming speed of 43.7 mm/s and a maximum self‐rotational angular velocity of 4.7 rad/s. When subjected to lateral water flow with a velocity of 150 mm/s, the robot stabilizes within 1.3 s. Furthermore, the robot can swim directionally into narrow spaces and accurately measure the pH value of the target water, highlighting its potential application in water environment monitoring and related scenarios.

## Graphical abstract

Inspired by the stable rotational descent of seeds in nature, this study designs a magnetic‐driven self‐rotation‐stabilized miniature swimming robot (MSMSR). Based on a resonance‐driven strategy, the robot exhibits high swimming performance, offering enhanced directional swimming and improved resistance to water flow disturbances compared to existing miniature swimming robots, highlighting its potential application in real‐world complex water environment monitoring and related scenarios.

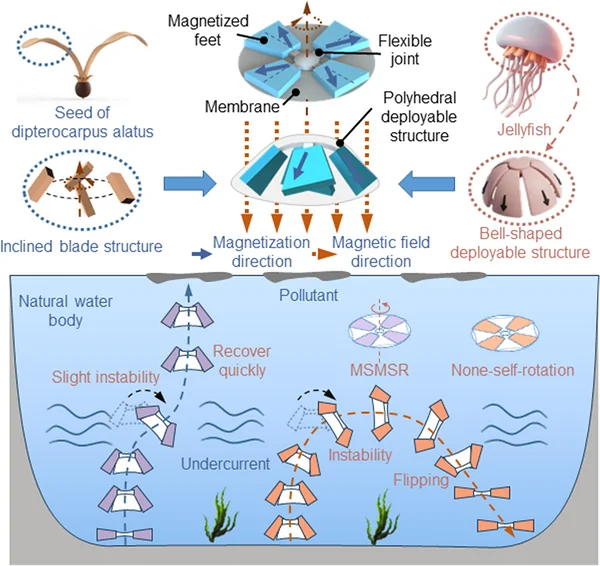

## 1 Introduction

In recent years, with the rapid development of materials science and intelligent manufacturing technologies, miniature swimming robots (MSRs) have become a prominent research focus at leading research institutions worldwide [1, 2, 3, 4, 5, 6, 7, 8, 9]. MSRs, a form of robot operating in underwater liquid media, have significant potential for applications in fields such as environmental monitoring due to their unique compliance and excellent environmental adaptability [10, 11]. Currently, MSRs employ various actuation methods, including pneumatic actuation [12, 13], dielectric elastomer (DE) actuation [14, 15], motor actuation [16, 17], shape memory alloy (SMA) wire actuation [18, 19], and light actuation [20]. Although these actuation methods have achieved some success in basic motion control, they still face several common challenges. For instance, pneumatic actuation, DE actuation, motor actuation, and SMA wire actuation are all wired actuation modes, and these robots tend to have relatively large and complex structures, with manufacturing processes that are generally difficult, making them unsuitable for narrow spaces in complex natural aquatic environments (see the top of Table S1). While light actuation has achieved wireless control to some extent and helped reduce the robot's size, the propagation of visible light in water is highly limited. Additionally, the lack of appropriate actuation devices means that research on this method is still in its early stages. In contrast, magnetic‐driven MSRs offer advantages such as lightweight structure, low operational noise, simple manufacturing process, and easy implementation of wireless controllability [21, 22, 23, 24]. Moreover, the gradual development of mobile electromagnetic systems [25, 26] in recent years has further expanded their application potential in real underwater environments, making them particularly suitable for scenarios such as pipeline leakage detection and exploration of confined underwater spaces [27, 28, 29].

Inspired by the motion patterns of microorganisms and aquatic organisms, researchers have developed various magnetic‐driven MSRs by combining smart materials with micro/nano manufacturing technologies [30, 31, 32, 33, 34, 35, 36, 37, 38, 39, 40, 41, 42]. Inspired by the morphology and motion mechanism of sperm, Tan et al. designed a polymorphous sperm‐like magnetic microswimmer with an extremely small size of only 0.1 mm, which also possesses material encapsulation (drug delivery) capabilities [31]. Similarly, inspired by the tail wagging of swordfish, Ou et al. designed a swordfish‐like wireless millirobot capable of making turns with different radii [32]. This type of robot moves forward by swinging under the influence of a periodic magnetic field. The swimming principle is relatively simple, with strong maneuverability. However, its swimming stability is poor, and it tends to deviate from the intended trajectory. In addition, inspired by the natural design of bacterial flagella, Zhang et al. designed an artificial bacterial flagellum that has a comparable shape and size to its organic counterparts and can swim in a controllable fashion using weak applied magnetic fields [35]. Liu et al. proposed magnetic soft spiral microfiberbots with high steerability, reliable maneuverability, and multimodal shape reconfigurability to perform robotic embolization in submillimeter regions [36]. This type of robot moves forward in a spiral motion when driven by a rotating magnetic field. Its size is typically extremely small, and its movement speed is relatively slow. Moreover, some researchers, inspired by the efficient swimming patterns of jellyfish, have designed a series of MSRs with pulse propulsion swimming modes. Ren et al. designed a jellyfish‐like swimmer with multiple locomotion modes [39], while Ye et al. designed a liquid metal‐enabled biomimetic robotic jellyfish [41]. These biomimetic jellyfish MSRs exhibit excellent swimming performance, which can efficiently perform tasks such as water quality monitoring. However, despite the significant progress made in the diversity of swimming modes for many biomimetic magnetic‐driven MSRs, their resistance to interference still presents notable limitations in real aquatic environments (see the bottom of Table S1). In real water areas, the water flow contains irregular disturbances and undercurrents, which will seriously disrupt the trajectory of the MSRs and the stability of its movement, preventing it from reaching the intended target effectively.

In this paper, we design a magnetic‐driven self‐rotation‐stabilized miniature swimming (MSMSR) inspired by the structural characteristics of the seeds of *Dipterocarpus alatus* and jellyfish. The main body is a polyhedral deployable structure (PDS) with flexible joints and four inclined magnetized feet. Flexible joints enable greater energy accumulation for rapid swimming, while the PDS facilitates pulsating propulsive swimming. Additionally, the inclined magnetized feet support rotational movement, collectively allowing the robot to achieve stable self‐rotation swimming. By investigating the coupling relationship between the robot's natural frequency and the driving magnetic field frequency, a resonance‐driven strategy is introduced to enhance the robot's swimming performance. The maximum swimming speed of the robot reaches 43.7 mm/s, and the maximum self‐rotational angular velocity reaches 4.7 rad/s. Moreover, our MSMSR exhibits excellent directional swimming capability and can quickly recover to a balanced state within 1.3 s even under lateral flow velocities as high as 150 mm/s, exhibiting significantly superior directional stability and resistance to flow interference. Further functional tests show that the MSMSR can perform point‐to‐point movement in narrow spaces and detect the pH value of water in target areas, highlighting its potential value in real‐world applications such as underwater environmental monitoring and pollution detection.

## 2 Results

### 2.1 Structure Design

**FIGURE 1:**
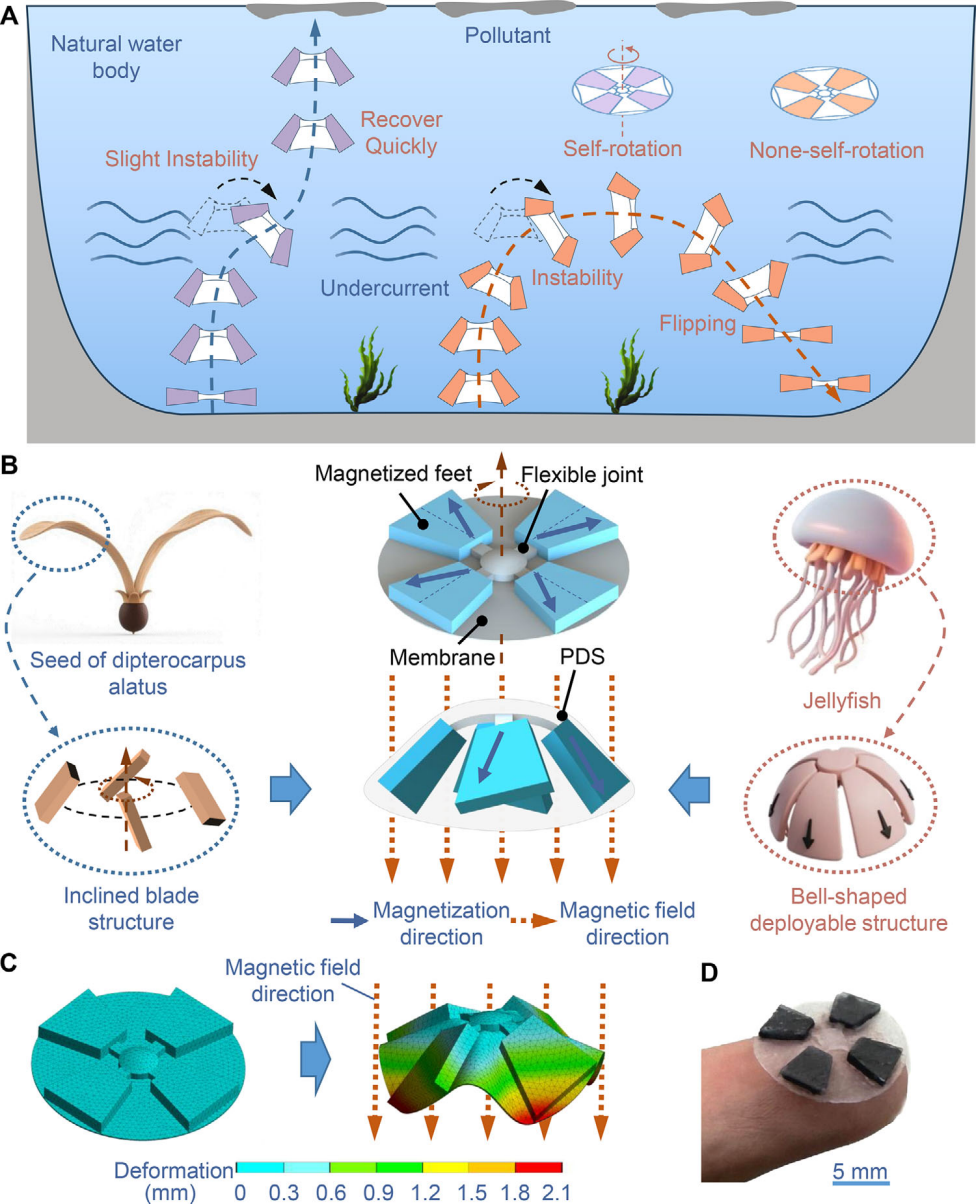
Structure design of MSMSR. (A) Schematic diagram of the different response processes of MSMSR and non‐self‐rotation MSR when swimming in a natural water body under the influence of undercurrent disturbances. (B) Bionic structure design of MSMSR, which is inspired by the seeds of *Dipterocarpus alatus* and jellyfish. (C) Deformation simulation diagram of the inclined flapping motion of MSMSR. (D) Physical prototype of MSMSR.

In natural water bodies, irregular perturbations and undercurrents are inevitable, making the environment far more complex than static water environments. Such complexity severely affects the movement trajectory and motion stability of swimming robots. This impact is more pronounced for MSRs, as they have weaker anti‐interference capabilities and are more susceptible to underwater perturbations and undercurrents. Most existing magnetic‐driven MSRs are designed for static water environments and therefore lack self‐rotation capabilities. When exposed to water flow impacts, they tend to experience instability and tilting, which ultimately result in functional damage and operational failure. Based on the law of conservation of momentum, rotating objects exhibit greater motion stability, with typical examples including fired bullets and the seeds of *Dipterocarpus alatus*. Inspired by this phenomenon, a magnetic‐driven self‐rotation‐stabilized MSR was developed to enhance the robot's motion stability, allowing it to better adapt to the complex conditions of real aquatic environments, particularly in resisting underwater water flow impacts. This MSMSR exhibits efficient directional swimming capability with strong resistance to water flow interference (Figure 1A).

In nature, when the seeds of *Dipterocarpus alatus* descend, they utilize their angled blades to guide airflow and generate rotational torque through interaction with the wind, thereby enabling self‐rotational movement. During the falling process, this self‐rotation helps the seeds resist lateral disturbances by conserving angular momentum, ensuring they do not flip over during descent [43, 44]. Drawing on the structure of the inclined blades of the seeds of *Dipterocarpus alatus*, an inclined blade structure similar to that of a bamboo dragonfly was designed. This structure generates a rotational torque by guiding water flow, driving the robot to rotate, thereby achieving stable self‐rotation swimming and effectively resisting the impact of lateral water flow (Figure 1B). The inclined blade structure enables the robot to rotate but does not allow it to swim rapidly. Jellyfish generate thrust by rhythmically contracting their bell‐shaped bodies to expel water and flapping these bells, which is one of the most efficient forms of swimming [45]. The bell‐shaped deployable structure is essential for jellyfish so that it can enclose and close its body while contracting, thereby generating thrust through the interaction with the surrounding water flow. Therefore, the inclined blade structure and the bell‐shaped deployable structure are integrated together to ensure that the robot can rotate and achieve rapid swimming. Moreover, flexible joints are introduced in the structural design to enable greater energy accumulation for rapid swimming [46, 47, 48].

The main body of the swimming robot is a PDS with flexible joints and four inclined magnetized feet (see center part of Figure 1B), which is designed as a centrally symmetric structure. Moreover, the robot's magnetic legs are arranged in a horizontally magnetized inclined configuration, rather than a vertical arrangement, for easier manufacturing. Additionally, during swimming, the robot generates thrust by flapping the magnetic legs to paddle the water, and the gap between the magnetic legs would reduce the swimming performance. Therefore, the gaps between the magnetic legs are filled with a soft membrane to assist in generating greater thrust. The magnetic legs are made from a silicone rubber base material, uniformly doped with neodymium‐iron‐boron (NdFeB) powder to achieve magnetic responsiveness. The magnetization direction of each magnetic leg is inclined at an angle *ψ* relative to the central symmetry axis, and they are connected through flexible silicone rubber joints. Thus, under the action of a vertical magnetic field, the robot will flap its magnetic feet obliquely downward (Figure 1C). When the four magnetic feet flap simultaneously, they generate an upward propulsive force and a torque that rotates along the robot's central axis, causing the robot to swim upward while rotating around its own axis. We fabricated the robot prototype using the method of micro‐injection molding, demolding, and micro‐assembly (Figure S1). The physical prototype of the robot is shown in Figure 1D. The body length (BL) of the robot is 15 mm (refers to the diameter of the membrane), with a thickness of 0.5 mm and a weight of 0.156 g, and the magnetization angle *ψ* of each magnetic leg is 30°.

### 2.2 Locomotion Principle and Actuation Strategy

**FIGURE 2:**
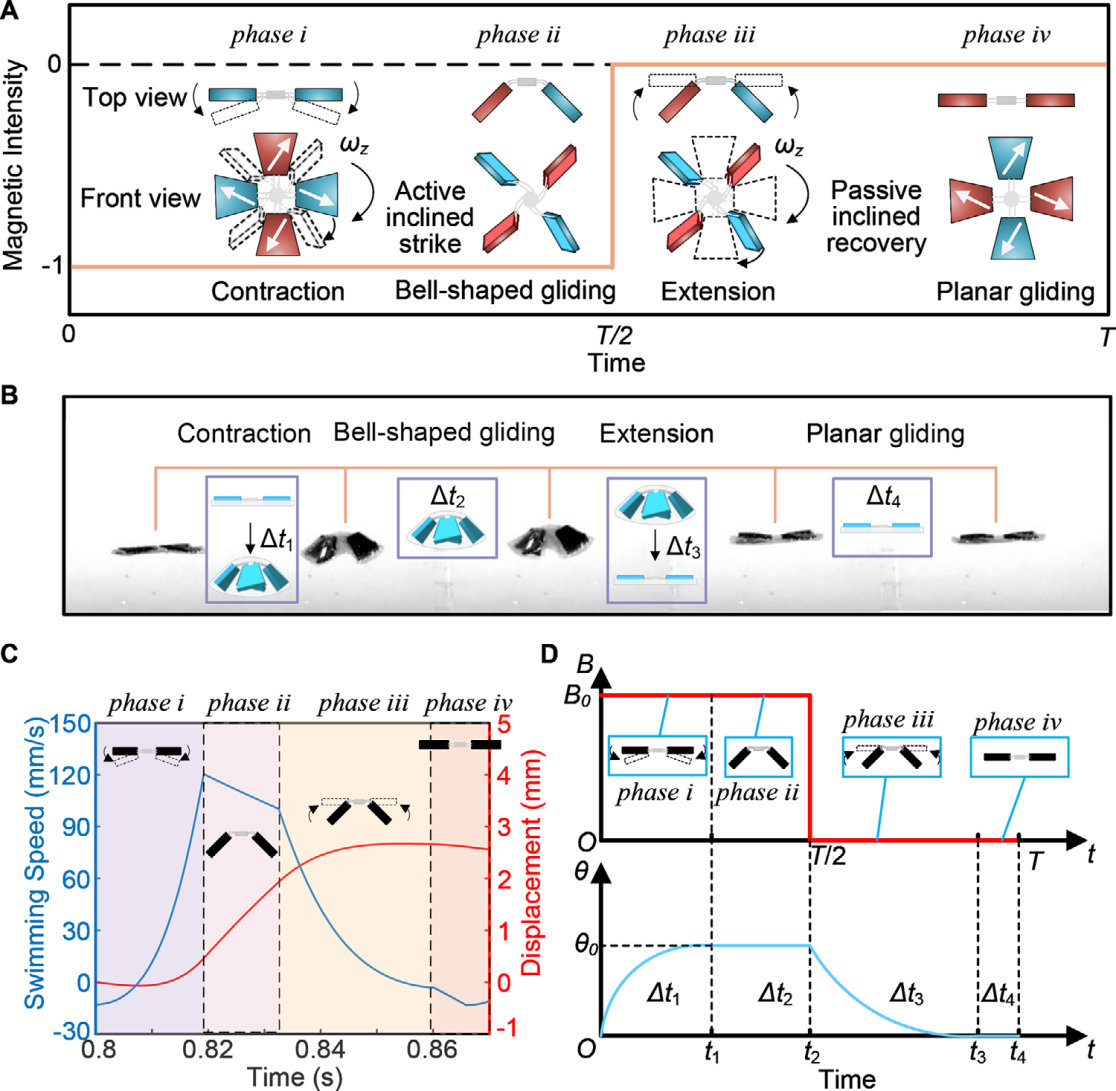
Self‐rotation locomotion gait analysis of MSMSR. (A) Driving signal and four phases (contraction, bell‐shaped gliding, extension, planar gliding) of a single self‐rotation locomotion cycle of MSMSR. (B) Swimming process of MSMSR during a single self‐rotation locomotion cycle. (C) Theoretical swimming speed and displacement prediction of the MSMSR's single self‐rotation locomotion cycle under a 15 Hz magnetic field. (D) Condition where all four phases of the MSMSR are present at high magnetic field frequencies.

To achieve the pulse propulsion and self‐rotational stability compound swimming capability of the MSMSR, a square magnetic field driving signal is applied to the designed robot (Figure 2A). Under the influence of the external magnetic field, the motion cycle of the robot is divided into four phases: *contraction*, *bell‐shaped posture gliding*, *extension*, and *planar posture gliding*. The specific times corresponding to each of these four stages are defined as Δ*t*_1_ to Δ*t*_4_. Initially, the robot is driven by the external magnetic field in the *Z*‐axis, causing the four magnetic legs to perform a paddling motion, striking the fluid medium. Through the reaction force from the fluid medium, the robot gains the thrust needed for swimming and self‐rotation. During this process, the robot gradually contracts and deforms from a planar posture to a bell‐shaped posture (*phase i: contraction*). When the robot reaches its maximum contracted state, but the *Z*‐axis magnetic field signal has not yet changed, the robot maintains the bell‐shaped posture and glides upward for a period of time (*phase ii: bell‐shaped posture gliding*). As the external magnetic field strength decreases to zero, the elastic potential energy stored in the flexible joints begins to be released, causing the four magnetic legs to swing in the opposite direction. This generates thrust opposite to the swimming and self‐rotational directions, rapidly reducing the robot's swimming speed and self‐rotational angular velocity. During this process, the robot gradually extends from the bell‐shaped posture to the planar posture (*phase iii: extension*). Finally, the robot fully recovers to the planar posture, but before entering the next cycle, it continues to glide in the planar posture under the influence of inertia (*phase iv: planar posture gliding*). A high‐speed camera was used to record the robot's motion process over one cycle, with an image acquisition frame rate of 1000 Hz (Figure 2B). It can be observed that the robot's motion posture within one cycle is consistent with the designed posture.

Based on the four motion phases of the MSMSR described above, we developed a theoretical swimming model for the robot under different phases (Note S1). The model reveals that the primary factors influencing the robot's locomotive performance include its structural dimensions and drag coefficient, etc. Furthermore, the swimming speed is closely related to the duration of each phase; that is, it also depends on the frequency of the driving signal. To investigate this relationship, we analyzed the swimming speed across various frequencies based on the theoretical model (Figure 2C and Figure S2). The results indicate that the speed varies significantly with the frequency, which can be attributed to the coupling between the robot's dynamic response and the driving magnetic field. Consequently, we investigated the relationship between the driving signal period and the phase durations. Figure 2D illustrates the case in which all four phases are present (other cases are shown in Figure S3), and the relationship generally holds:

$$T/2=\Delta t_{1}+\Delta t_{2}=\Delta t_{3}+\Delta t_{4}$$

where *T* is the period of the magnetic field driving signal. From the theoretical model, it can be found that the second and fourth phases reduce the swimming speed. Therefore, minimizing the duration of these two phases (Δ*t*_2_ and Δ*t*_4_) is critical for enhancing the robot's speed.

As the magnetic field frequency increases, the durations of the second phase (Δ*t*_2_) and the fourth phase (Δ*t*_4_) gradually decrease. At the critical condition Δ*t*_4_ = 0, the robot achieves its maximum swimming speed. At this point, since Δ*t*_1_≈Δ*t*_3_, the robot's natural frequency *f_r_* = 1/(Δ*t*_1_+Δ*t*_3_) coincides with the driving frequency *f_d_* = 1/*T*. With a further increase in frequency, the magnetic legs cannot fully extend to the planar posture within one swimming cycle, resulting in a decrease in swimming speed as the frequency continues to rise. This indicates that the robot achieves maximum swimming speed when the drive frequency coincides with the robot's resonant frequency. However, in actual swimming, factors such as water resistance cause the downward swing speed of the magnetic legs to be faster than the rebound speed, meaning Δ*t*_1 _< Δ*t*_3_. Consequently, the robot's peak swimming speed does not occur exactly at the resonance point (*f_d_*  = *f_r_*), but rather within a specific range of driving frequencies, that is, 1/(2Δ*t*_3_) < *f_d_* < 1/(2Δ*t*_1_).

In addition, we established a self‐rotation dynamics model for the robot (Note S2). Similarly, the self‐rotational motion can be divided into four phases. The model indicates that the robot's self‐rotation is governed by the circumferential force *F_c_* and the circumferential hydrodynamic drag *D_c_*. Increasing either the velocity at which the robot's paddling motion interacts with the surrounding fluid during propulsion or the paddling amplitude can increase the robot's self‐rotational angular velocity.

### 2.3 Locomotion Performance

**FIGURE 3:**
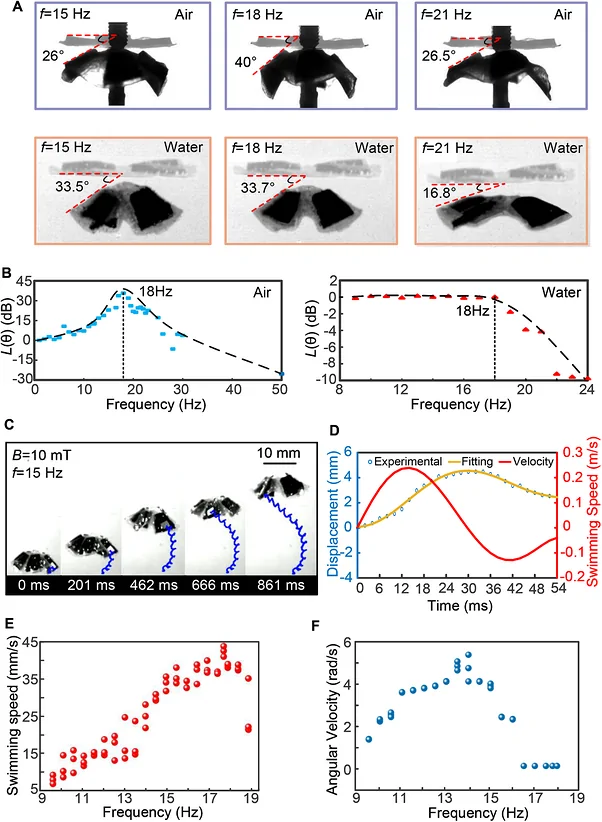
Experimental results of MSMSR about resonance frequency and locomotion performance. (A) Oscillation of MSMSR in air (top) and water (bottom) at different magnetic field frequencies (15, 18, 21 Hz). (B) Magnetic legs' oscillation amplitude of MSMSR in air (left) and water (right). (C) Locomotion process of MSMSR under the 10 mT, 14 Hz driving magnetic field. (D) Instantaneous swimming speed and displacement of MSR during a single locomotion cycle under the 10 mT, 15 Hz driving magnetic field. (E) Relationship between the driving magnetic field frequency and the MSMSR's average swimming speed. (F) Relationship between the driving magnetic field frequency and the MSMSR's self‐rotational angular velocity.

Although the robot in this paper is made of soft materials, it still exhibits resonance phenomena. Based on the Lagrangian equation, we established a dynamic response model for the flexible joint of the developed robot (Note S3). Under the action of a uniform external magnetic field, the robot's magnetic leg is subjected to a bending moment, so the flexible joint can be regarded as a cantilever beam structure with one end fixed and the other end whose angle and position change with the rotation angle. We used an electronic universal testing machine (INSTRON) to measure the Young's modulus of both the magnetic leg and the nonmagnetic joint (Figures S4 and S5), and we calculated the first‐order natural frequency of the flexible joint cantilever beam model to be 17.6 Hz. To characterize this resonance, we tested the robot's resonant frequency in both water and air (Figure S6). Under magnetic field driving at different frequencies, the swing angles of the robot's magnetic feet were recorded using a high‐speed camera (Figure 3A). Through image processing, we obtained the frequency‐domain and time‐domain response characteristics of the robot and constructed the Bode plot of the magnetic legs’ oscillation amplitude (Figure 3B). According to the Bode plot, the robot's natural frequency in both water and air is 18 Hz, which is close to the theoretical calculation results.

To investigate the influence of the driving magnetic field frequency on swimming speed, the locomotion of the robot under various magnetic field frequencies was recorded using a high‐speed camera (Movie S1), and its motion trajectories were extracted via marker point recognition (Figure 3C and Movie S2), from which the instantaneous swimming speed and displacement over one cycle were computed. It was found that under the driving magnetic field of 10 mT at 15 Hz, the robot's step displacement was 2.42 mm, and the swimming speed was 36.7 mm/s (Figure 3D). Under this condition, the robot's motion cycle was prolonged, and the duration of liquid resistance was increased, causing the minimum instantaneous speed to become negative and thereby resulting in noticeable backward movement during swimming. Under the driving magnetic field of 10 mT at 17.8 Hz (around the resonance frequency), the robot's step displacement increased to 2.46 mm, and the swimming speed increased to 43.7 mm/s (Figure S7). At this point, the robot's speed loss from the planar posture gliding stage was minimized, and the backward movement was significantly suppressed. Under the driving magnetic field of 10 mT at 18.5 Hz, the robot's motion amplitude decreased, with a step displacement of only 2.09 mm and a swimming speed of 38.7 mm/s (Figure S8). The results indicate that the robot's swimming speed first increases and then decreases as the external magnetic field frequency increases, which is consistent with the theoretical model established earlier. Figure 3E shows the relationship between the frequency of the driving magnetic field and the average swimming speed of the robot. It can be clearly found that when the frequency of the driving magnetic field is near the robot's resonant frequency (17.8 Hz), the robot's swimming speed reaches a maximum value of 43.7 mm/s.

Correspondingly, the frequency of the driving signal also affects the robot's self‐rotational angular velocity. However, since both the position and orientation change significantly during rotation, it is difficult to obtain the instantaneous rotation angle and angular velocity solely through a visual approach. Nevertheless, the average self‐rotational angular velocity can be determined, and its relationship with the driving magnetic field is shown in Figure 3F. It can be observed that as the magnetic field frequency increases, the robot's self‐rotational angular velocity first increases and then decreases; in contrast to the swimming speed, the maximum rotation angular velocity does not occur near the resonance frequency but instead at 14 Hz. When the magnetic field frequency is near the robot's resonance frequency (17.8 Hz), the rotation angular velocity approaches zero. This is because, when the driving frequency is below 14 Hz, increasing the frequency raises the flapping frequency of the robot against the water and shortens the duration of each motion cycle, thereby continuously increasing its self‐rotational angular velocity. In contrast, when the driving frequency exceeds 14 Hz, the backward rotation angle generated during the extension process gradually increases, which imposes a stronger inhibitory effect on the self‐rotation. As the magnetic field frequency further approaches the resonance frequency of the robot, the forward rotation angle generated during the propulsion process is progressively canceled out by the backward rotation angle generated during the extension process. As a result, the robot only oscillates left and right within a small range instead of achieving sustained self‐rotation. This phenomenon of a step‐out frequency for self‐rotation is also consistent with the related trends reported in existing studies [49, 50]. Therefore, to balance the rotation and swimming of the robot, the frequency range of the driving magnetic field can be selected between 13 and 14 Hz.

In addition, we also fabricated two control robots with magnetization angle *ψ* of 15° and 45° (Figure S9), denoted as MSMSR‐1 and MSMSR‐2, respectively, and carried out corresponding experiments to investigate the effect of magnetization angle on the robot's rotational angular velocity (Figures S10 and S11). Based on the results shown in Figure 3F and Figure S12, it can be concluded that, within the 0°–45° range, the differences in peak rotational angular velocity among robots with different magnetization angles are relatively small, remaining within 10% overall. In contrast, a more pronounced trend is that, as the magnetization angle increases, the driving frequency corresponding to the peak rotational angular velocity gradually decreases. Specifically, for magnetization angles of 15°, 30°, and 45°, the corresponding frequencies are 17, 14, and 12 Hz, respectively. These results indicate that, in future designs, robots with appropriate magnetization angles may be selected according to the target driving magnetic field frequency range, thereby enabling effective tuning of their rotational performance.

### 2.4 Directional Swimming Performance

**FIGURE 4:**
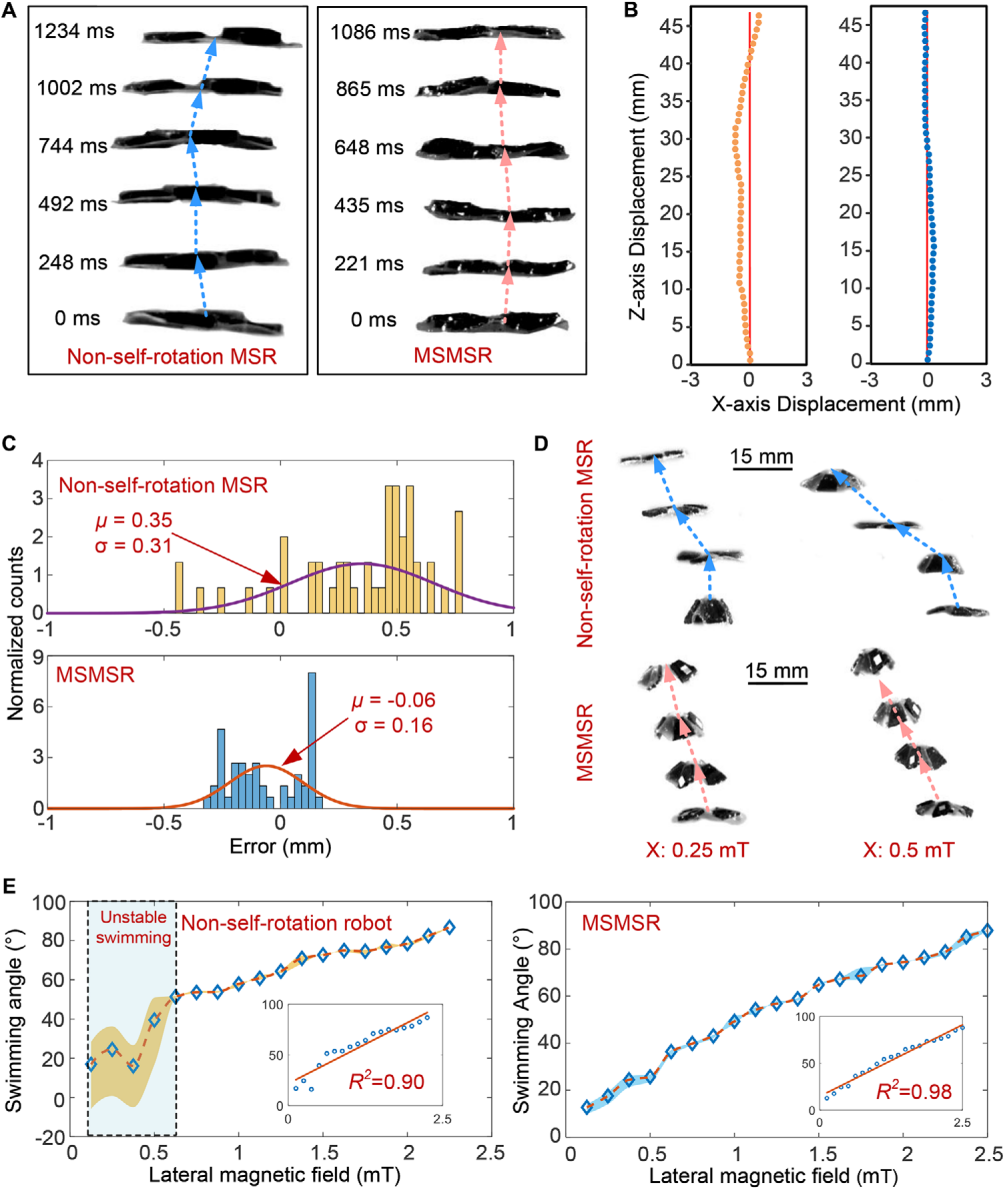
Experimental results of MSMSR about directional swimming performance. (A) Vertical swimming process of non‐self‐rotation MSR (left) and MSMSR (right). (B) Center position coordinates for vertical swimming of non‐self‐rotation MSR and MSMSR. (C) Normalized distribution of the center position coordinates for vertical swimming of non‐self‐rotation MSR and MSMSR. (D) Inclined swimming process of non‐self‐rotation MSR (top) and MSMSR (bottom). (E) Relationship between inclined swimming angle and lateral magnetic field of non‐self‐rotation MSR and MSMSR (The inset shows a comparison of linearity).

Directional swimming capability is an important performance indicator for MSR, as it reflects the accuracy of the robot's movement. The MSMSR proposed in this study exhibits favorable directional swimming capability, as it can maintain the stability of its central axis through self‐rotation. We tested the vertical swimming performance of both the MSMSR and a non‐self‐rotating MSR (same geometric parameters) with the same magnetic field condition (14 Hz, 10 mT); their swimming postures and positions are illustrated in Figure 4A and Movie S3. The non‐self‐rotating MSR swings left and right when swimming upward. This phenomenon occurs because the robot's swimming generates small irregular water currents around it, which in turn affect the robot's posture. In contrast, our MSMSR is not disturbed by such small water currents and can still maintain linear movement. Figure 4B,C shows the central positions of the robots during swimming, obtained via image processing. It can be seen that the MSMSR has a mean error of −0.06 mm and a standard deviation of 0.16 mm for its central position, which are significantly smaller than those of the non‐self‐rotation MSR, with a mean error of 0.35 mm and a standard deviation of 0.31 mm. This indicates that the robot maintains a small lateral offset during vertical swimming, demonstrating high vertical directional stability. Therefore, it can be concluded that self‐rotational motion significantly enhances the robot's stability in vertical motion.

We also tested the robot's directional swimming performance during inclined swimming, i.e., when the robot is subjected to magnetic fields in both the *X*‐ and *Z*‐axis directions simultaneously. In these experiments, the vertical magnetic field was fixed at 14 Hz and 10 mT, and the lateral magnetic field was a DC magnetic field. The robot's motion trajectories were recorded using a high‐speed camera (Figure 4D and Movie S4). Similarly, the MSMSR has a more stable swimming trajectory and better directional swimming performance. Furthermore, we investigated the effect of lateral magnetic fields on the directional swimming of the robot, and the relationship between the inclined swimming angle and the lateral magnetic fields (*X* axis) for the non‐self‐rotation MSR and the MSMSR was obtained (Figure 4E). Here, the inclined swimming angle is defined as the angle between the vertical direction and the line connecting the geometric center of the robot at its initial state and that when it first reaches the water surface. When the lateral magnetic field is relatively small (< 0.5 mT), the inclined swimming angle of the non‐self‐rotation MSR fluctuates significantly; however, such fluctuations do not occur when the lateral magnetic field is relatively large. This is because a larger lateral magnetic field exerts a more dominant influence on the robot's motion, thereby minimizing the impact of disturbances. In contrast, when the lateral magnetic field is weak, the robot is more susceptible to the effects of disturbances. This phenomenon does not occur during the swimming of the MSMSR, which can maintain stable swimming even when the lateral magnetic field is small. In addition, compared with the non‐self‐rotation MSR, the MSMSR demonstrates a better linear relationship between the inclined swimming angle and the lateral magnetic field (see the inset of Figure 4E). Specifically, the *R* ^2^ for the self‐rotation robot is 0.98, whereas that for the non‐self‐rotation robot is 0.9. This finding indicates that the proposed MSMSR is capable of achieving precise directional swimming over a wide angular range (−87.4°to 87.4°), especially at small inclined swimming angles (−25.58°to 25.58°), thereby laying a solid foundation for the subsequent control of the robot (Figure S13). In addition, we further investigated the effect of the vertical magnetic field frequency on the robot's swimming angle (Figure S14). The results show that, under otherwise identical conditions, the swimming angle of the MSMSR decreases with increasing vertical magnetic field frequency, because the higher swimming speed shortens the exposure time to the lateral magnetic field.

### 2.5 Stability Performance

**FIGURE 5:**
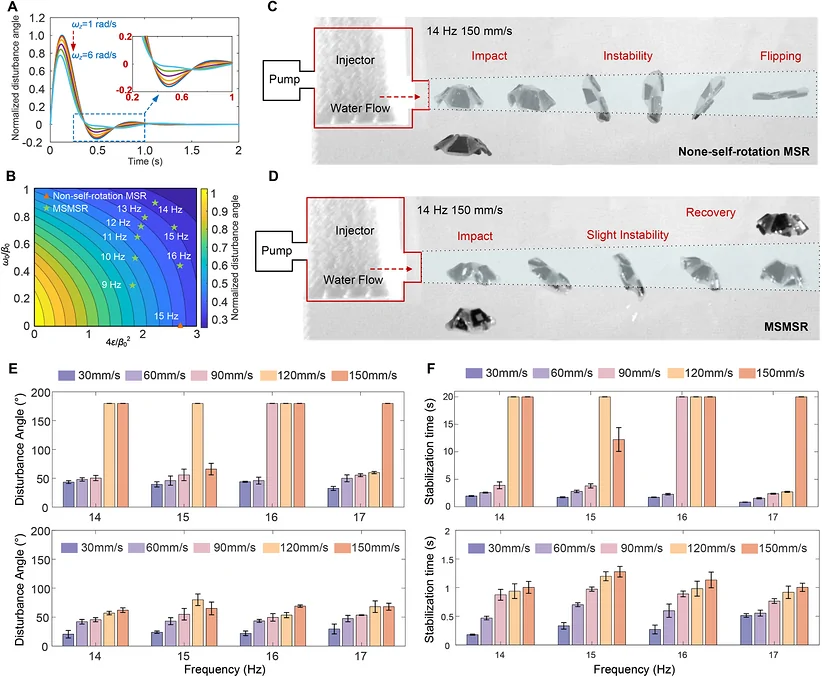
Experimental results of MSMSR about stability performance. (A) Predicted dynamic response of MSMSR to impacts at different self‐rotational angular velocities. (B) Normalized maximum disturbance angle of non‐self‐rotation MSR and MSMSR at different frequencies (closer to the upper right indicates stronger robot stability). (C) Response of the MSMSR after lateral water‐flow impact. (D) Response of the non‐self‐rotation MSR after lateral water‐flow impact. (E) Disturbance angle of the MSRs under different water flow velocities, where the top is the non‐self‐rotation MSR and the bottom is the MSMSR. (F) Stabilization time of the MSRs under different water flow velocities, where the top is the non‐self‐rotation MSR and the bottom is the MSMSR.

Water flow disturbances can cause significant interference to the movement of MSRs, and thus the ability to resist water flow disturbances has become an important dimension for evaluating the performance of MSRs. To investigate the mechanism by which self‐rotation enhances a MSR's ability to resist water flow disturbances, a theoretical analysis model (Note S4) for the MSR's self‐rotation stability is established based on the model proposed by Kim et al. [43]. In the model, the decoupled oscillation equation is as follows.

(1)

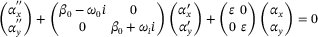

where *α_x_* and *α_y_* are the disturbance angle after decoupling, *β*_0_ = *πμr* ^2^/8*I_x_* Re*_B_*, *ω*_0_ = (*I_z_*‐*I_y_*)/*I_x_ω_T_*, *ε* = *Wd*/*I_x_*, *μ* is the dynamic viscosity of water, *r* is the radius of robot, *ω_T_* is the rotational angular velocity of the robot's rotation along the *Z* direction, Re*_B_* is the Reynolds number in water, *W* is the work done by the magnetic force on the robot, *I_x,y,z_* are the moment of inertias for directions *X*, *Y* and *Z*, and *d* is the distance between the center of gravity and the center of pressure. By solving the differential equation, the relationship between the robot's disturbance angle and time can be obtained as follows:

(2) $$\Lambda \left(t\right)=\sum_{i=1}^{4}C_{i}e^{\lambda_{i}t}$$

where *C_i_* is a constant, and *λ_i_* is the eigenvalue corresponding to the robot's different swimming speeds and rotational angular velocities. Figure 5A predicts the variation of the disturbance angle over time for the robot at different rotational angular velocities. It is evident that as the parameter *ω_T_* increases, the amplitude of the robot's maximum disturbance angle monotonically decreases. We define that a robot can be considered to have recovered stability after being affected by disturbances when the disturbance angle error is less than ± 0.05. The relationship between the robot's stabilization time and rotational angular velocity is obtained (Figure S15). It can be seen that as the rotational angular velocity increases, the robot's recovery time decreases, demonstrating that the greater the robot's rotational velocity, the stronger its ability to resist water flow disturbances.

To further investigate the combined effect of self‐rotational angular velocity and swimming speed on the stability indicators, we established the relationship between the disturbance angle and the stability parameters *ω*_0_/*β*_0_ and 4*ε*/*β*_0_^2^ based on the model (Figure 5B), where the parameters *ω*_0_/*β*_0_ and 4*ε*/*β*_0_^2^ are positively correlated with the robot's angular velocity and swimming speed, respectively (Figure S16). Besides, the disturbance angle was normalized using the min‐max normalization method, and the magnitude of the robot's normalized disturbance angle was defined as 1 when both its swimming speed and rotational velocity were zero. Moreover, the swimming speed and self‐rotational angular velocity of the robots used in Figure 5B are the data measured experimentally in the previous section. As shown in Figure 5B, the disturbance angle generated by the MSMSR after being disturbed first decreases and then increases as the driving field frequency increases. This is because when the frequency exceeds 14 Hz, the negative effect of the decrease in the robot's rotational velocity on stability outweighs the positive effect brought by the increase in swimming speed. Therefore, the disturbance angle reaches its minimum value when the driving frequency is 14 Hz, at which point the robot exhibits the most stable movement. In addition, under the same driving frequency, the disturbance angle generated by the MSMSR is smaller than that of the non‐self‐rotation MSR. It can thus be concluded that self‐rotation effectively enhances the robot's ability to resist disturbances.

To investigate the anti‐disturbance effect of the developed MSMSR, we conducted lateral water flow impact experiments on the MSMSR driven by magnetic fields of different frequencies. The experiments set five water flow velocities: 30, 60, 90, 120, and 150 mm/s, where the lateral water flow was generated by an injector driven by a syringe pump. Meanwhile, non‐self‐rotation MSR were used as the control group, and tests were carried out under exactly the same experimental conditions. We used a high‐speed camera to record the robot's posture and trajectory after being impacted by the water flow (Movie S5). Figure 5C,D shows the response process of both the MSMSR and non‐self‐rotation MSR after being impacted by lateral water flow with the same magnetic field frequency (14 Hz) and the same flow velocity (150 mm/s). The MSMSR exhibited slight instability after being impacted by the water flow and quickly recovered to stable swimming. In contrast, the non‐self‐rotation MSR experienced greater instability after the impact, eventually flipping over and being unable to continue swimming. Based on the MSRs’ motion movies, we extracted the robot's disturbance angle after the disturbance and calculated the time it takes to recover stability (Figure 5E,F). Among them, a disturbance angle of 180° indicates that the MSR has flipped, and a stabilization time of 18 s indicates that the MSR is unable to return to a stable state. The results demonstrate that the disturbance angle of the MSMSR is generally smaller than that of the non‐self‐rotation MSR, and it consistently recovers stability across all experiments, which can be attributed to the self‐rotational motion. As the impact water flow velocity increases, the disturbance angle of the self‐rotation increases, and the stabilization time also becomes longer. For the MSMSR, the maximum disturbance angle is approximately 80°, and it can always recover stability within 1.3 s. In contrast, the non‐self‐rotation MSR is more susceptible to flipping at higher water flow velocities (> 120mm/s) and fails to recover stability, thereby losing its operational capability. In addition, we also tested the performance of MSMSR‐1 and MSMSR‐2 under lateral water flow impacts. The results in Figures S17 and S18 show that both MSRs with different magnetization angles exhibited excellent overall disturbance‐resistance performance, with no loss of stability observed during the experiments and stabilization times all below 2.5 s. Moreover, both MSRs showed the smallest disturbance angle and the fastest recovery when their self‐rotational angular velocity reached the maximum. These experimental findings confirmed that self‐rotational motion significantly enhances the robot's ability to resist lateral water flow disturbances. Furthermore, by appropriately tailoring the robot's magnetization angle, stable swimming can be achieved over different driving‐frequency ranges.

In addition, to more comprehensively evaluate the anti‐disturbance capability of the MSMSR, we further conducted 120 mm/s water flow impact experiments on both the MSMSR and the non‐self‐rotation MSR under different driving frequencies, with the flow applied at upward 15° and downward 15° inclinations (Figure S19). The results in Figures S20 and S21 show that, regardless of whether the robot was subjected to upward‐inclined or downward‐inclined water flow impacts, the MSMSR consistently exhibited a smaller disturbance angle and a shorter recovery time than the non‐self‐rotation MSR at different frequencies. These results further demonstrate that self‐rotational motion can effectively enhance the overall locomotion robustness of the robot.

### 2.6 Water Quality Detection of Target Area

**FIGURE 6:**
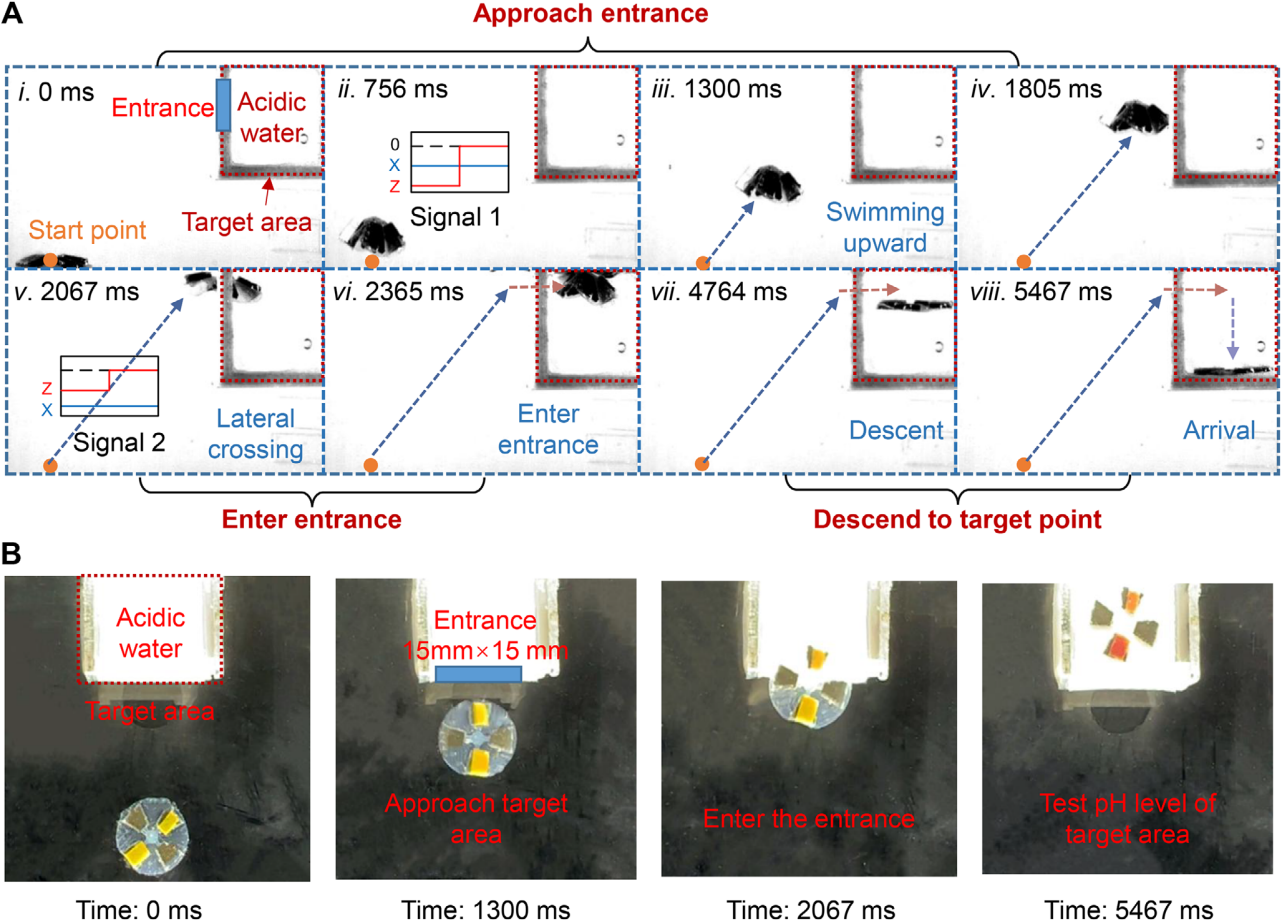
Process of the proposed MSMSR swimming to the target area and monitoring water quality. (A) Side view, (*i–iv*) Applying signal 1, the MSMSR performs inclined swimming to approach the entrance. (*v, vi*) Applying signal 2, the MSMSR performs lateral swimming to enter the entrance. (*vii, viii*) Turning off the magnetic field, the MSMSR descends to the target point and performs pH detection. (B) Top view: After the MSMSR reaches the acidic area, the pH test paper it carries turns red, demonstrating its water quality detection capability.

As mentioned above, the MSMSR developed in this study exhibits excellent performance in both directional swimming capacity and resistance to water flow disturbances, which can meet the requirements of task scenarios that demand precise swimming in confined spaces. Figure 6A illustrates a scenario of pH detection in a target area within a narrow water space. A small space was set as the target detection point at the upper left corner of the water tank, with an entrance size of 15 × 15 mm^2^, which is approximately equal to the robot's body length. First, the robot was required to move directionally to the entrance (Figure 6A i–iv). Subsequently, a lateral magnetic field was applied to enable the robot to swim into the entrance (Figure 6A v–vi). Finally, the magnetic field was turned off, allowing the robot to stay in the target space for a certain period to perform the pH detection (Figure 6A vii–viii). Figure 6B presents a top view of the entire process, from which the color change of the pH test paper carried by the robot can be observed, thereby enabling the determination of the pH value at the target location (Movie S6). Besides, to verify the capability of the proposed MSMSR for detecting water temperature variations during locomotion, a local hot‐water region was generated in a water tank by injecting 60°C hot water into a 20°C cold‐water background using a syringe pump. The MSMSR integrated with a temperature‐sensitive sensor (turning red at 50°C) was initially placed in the cold‐water region and then magnetically driven toward the target area. As the robot entered the hot‐water region, the sensor turned red, indicating its response to the elevated temperature (Figure S22). These results demonstrate that the robot possesses good locomotion controllability and promising potential for monitoring multiple physicochemical properties of water bodies.

## 3 Discussion

In conclusion, we have successfully designed a MSMSR with enhanced pulse propulsion and self‐rotational stability composite motion capabilities. The proposed MSRSR features a PDS with flexible joints and four inclined magnetized feet, inspired by the rotation mechanism of the seeds of *Dipterocarpus alatus*. The design leverages flexible joints to facilitate energy accumulation for rapid swimming and the PDS structure for pulsating propulsion. The self‐rotational stability of the MSMSR is achieved through its inclined magnetized feet, allowing for stable self‐rotation swimming. By exploring the coupling relationship between the MSR's natural frequency and the external magnetic field frequency, we introduced a resonance‐driven strategy to optimize swimming performance. Experimental results demonstrate that the MSR achieves a maximum swimming speed of 43.7 mm/s and a self‐rotational angular velocity of 4.7 rad/s. Furthermore, the MSMSR exhibits superior directional swimming ability, maintaining stable directional swimming within a deflection range of ± 87.4°. When subjected to a lateral water flow impact of 150 mm/s, the MSRSR is able to swiftly regain stability within 1.3 s due to its self‐rotational capability. The MSMSR also shows promising functionality in narrow space navigation and pH value detection, highlighting its potential for applications in water environment monitoring and pollution detection.

Overall, this study provides new insights into the design of underwater magnetic‐driven MSRs. The proposed inclined magnetized flexible structure design principles are expected to guide the configuration optimization of other flexible motion devices and soft MSRs. While the robot's directional swimming and stability performance have been initially validated in a laboratory water tank, further application testing in real natural water bodies remains limited. Future work could further integrate a mobile electromagnetic actuation system with a miniaturized vision module to enable locomotion control and dynamic sensing in real underwater environments. As one feasible mobile deployment scheme, the Helmholtz coil module and camera could be integrated with the end effector of a six‐degree‐of‐freedom robotic arm, allowing the coil pair to follow the robot along the pipeline. The camera would continuously capture the position and motion state of the robot and provide feedback to the robotic‐arm control system. The position of the coil module could then be adjusted accordingly to maintain the robot within the relatively uniform effective magnetic‐field region near the center of the coil pair. This scheme could extend the effective actuation range and provide a potential approach for closed‐loop locomotion control and dynamic monitoring in confined underwater environments such as pipelines.

Meanwhile, the current sensing demonstrations remain proof‐of‐concept studies rather than a complete continuous monitoring system. Future work could integrate miniaturized pH and temperature sensors, low‐power signal‐conditioning circuits, and RF‐enabled wireless readout modules into the robot. The flexible body could also be fabricated from functional materials, such as pH‐responsive, temperature‐sensitive, or heavy‐metal‐ion‐sensitive hydrogels, to further integrate locomotion and sensing. Such developments may enable real‐time monitoring of water physicochemical parameters and expand the robot's potential applications in pipeline leakage detection and water‐quality monitoring in confined underwater environments.

## 4 Materials and Methods

### 4.1 Manufacturing Process

The MSMSR was fabricated using an injection molding process. The mold was made from polylactic acid (PLA) material, which was produced using high‐precision, high‐efficiency 3D printing technology. The robot's legs were constructed from a composite material made by mixing silicone rubber (Dragon Skin 30, Smooth‐On, USA) and NdFeB powder (average particle size of 5 µm) in a 1:1 mass ratio. The density of the composite material was 2.39 g/cm^3^. After the mixture was injected into the mold, it was cured in an oven at 45°C for approximately 30 min. The cured legs were then placed in a magnetization mold and fully magnetized in a strong magnetic field ranging from 1.6 to 3 T to ensure that they acquire a specific magnetization direction. During the assembly process, the legs were precisely positioned in the preset areas of the robot mold. They were fixed and bonded using specialized adhesive material (Dragon Skin 30, Smooth‐On, USA), and a film was produced. Sufficient time was allowed for complete curing. Finally, the fully cured robot was carefully removed from the mold using precision tools such as tweezers.

### 4.2 Construction of the Magnetic Driving System

The electromagnetic driving system consists of a magnetic control module and a visual monitoring module (Figure S23). The magnetic control module uses a three‐axis Helmholtz coil (PS‐3HM355, China), which generates a directionally adjustable and uniformly strong magnetic field in three‐dimensional space. The effective uniform magnetic field range is 80 mm × 56 mm × 40 mm. The magnetic field strength generated by the coil is approximately linearly related to the input current, allowing for stable control by adjusting the current. The driving signal is output by a signal generator (FY8300S, China) and amplified by a power amplifier (PS‐253D, China) before being input to the coil to produce the required magnetic field. To capture and feedback the robot's motion state, the visual module consists of a high‐speed camera (FASTCAM Mini UX100), which is installed in front of the robot's working space. It records the robot's position and posture during movement and transmits the data to the host computer for processing and analysis.

### 4.3 Robot Locomotion Testing

The robot's locomotion under the influence of external magnetic fields at different frequencies was recorded using a high‐speed camera (as mentioned above) at a rate of 1000 frames per second. The height of the robot at different time intervals was recorded and measured by frame extraction, from which the average swimming speed was calculated. Distinctive small markers with high color contrast were affixed to the robot's magnetic legs. Using trajectory recognition, the horizontal displacement of each marker's centroid was measured and converted from image distance to the actual rotation angle of the marked magnetic legs during self‐rotational swimming. The time elapsed was then calculated from the video frames, allowing for the calculation of the robot's self‐rotational angular velocity. Each experiment was conducted more than three times, and the average value was taken as the final result.

### 4.4 Stability Performance Testing

The stability performance of the robot was evaluated via lateral flow experiments at flow velocities of 30, 60, 90, 120, and 150 mm/s. The robot was subjected to a frontal impact while swimming under different frequency magnetic field drives. The water flow was provided by a syringe pump (Yuanhang Power Environmental Control Machinery Co., Ltd., Xinxiang, China), with the injector outlet connected to the experimental water tank via a 5 mm diameter rubber hose. The end of the hose was fixed to the side of the tank near the robot. When the robot moved close to the outlet of the rubber hose, the injector was quickly activated to release the water over a set period of time, generating a stable linear water flow impact on the robot. The entire impact process was recorded by a high‐speed camera (as mentioned above) at a rate of 1000 frames per second. Each impact experiment at different frequencies was repeated more than three times.

### 4.5 Targeted Water Quality Detection Testing

A U‐shaped structure block made of PLA with dimensions of 20 mm × 20 mm × 20 mm and an opening size of 15 mm × 15 mm was fabricated using 3D printing technology. After enclosing glass sheets on both sides of the structure, it was positioned at the upper‐left corner of the water tank, at the center of the Helmholtz coil. Before the experiment, pH test strips were cut into the same shape as the robot's magnetic legs and attached to their surfaces. During the experiment, a high‐speed camera (as mentioned above) was used to record at a rate of 1000 frames per second from the side of the water tank, while a smartphone simultaneously recorded from the top of the tank.

### 4.6 Determination of the Normalized Distribution of Center Position Coordinates

The normalized distribution of the center position coordinates was obtained from the vertical swimming experiments of robots. Specifically, the swimming motion was recorded by a high‐speed camera, and the geometric center of the robot at the initial position was defined as the reference point. The swimming trajectory was then discretized into multiple successive stages, and the geometric center coordinates at each stage were extracted sequentially. For each extracted point, the absolute lateral deviation from the theoretical vertical swimming path was calculated. The resulting deviation data were subsequently normalized and statistically analyzed to obtain the normalized distribution of the center position coordinates.

## Author Contributions

**Yanling Tian**: supervision, Writing – review and editing. **Chuhan Zhang**: methodology, investigation, visualization, writing – original draft. **Cunman Liang**: conceptualization, methodology, investigation, funding acquisition, project administration, writing – original draft. **Fujun Wang**: conceptualization, methodology, funding acquisition, project administration, supervision, writing – review and editing. **Hao Zhang**: conceptualization, methodology, investigation, visualization, writing – original draft.

## Conflicts of Interest

The authors declare no conflicts of interest.

## Supporting information




**Supporting File 1**: advs77382‐sup‐0001‐MovieS1.mp4.

**Supporting File 2**: advs77382‐sup‐0002‐MovieS2.mp4.

**Supporting File 3**: advs77382‐sup‐0003‐MovieS3.mp4.

**Supporting File 4**: advs77382‐sup‐0004‐MovieS4.mp4.

**Supporting File 5**: advs77382‐sup‐0005‐MovieS5.mp4.

**Supporting File 6**: advs77382‐sup‐0006‐MovieS6.mp4.

**Supporting File 7**: advs77382‐sup‐0007‐SuppMat.docx.

## Data Availability

All data needed to evaluate the conclusions in the paper are present in the paper and the Supplementary Materials. Additional data related to this paper may be requested from the corresponding author via email.

## References

[1] Y. Zhang , D. Kong , Y. Shi , et al., “Recent Progress on Underwater Soft Robots: Adhesion, Grabbing, Actuating, and Sensing,” Frontiers in Bioengineering and Biotechnology 11 (2023): 1196922, 10.3389/fbioe.2023.1196922. 37614630 PMC10442648

[2] J. Qu , Y. Xu , Z. Li , et al., “Recent Advances on Underwater Soft Robots,” Advanced Intelligent Systems 6 (2024): 2300299, 10.1002/aisy.202300299.

[3] S. M. Youssef , M. Soliman , M. A. Saleh , M. A. Mousa , M. Elsamanty , and A. G. Radwan , “Underwater Soft Robotics: A Review of Bioinspiration in Design, Actuation, Modeling, and Control,” Micromachines 13 (2022): 110, 10.3390/mi13010110. 35056275 PMC8778375

[4] L. Liu , X. Huang , X. Zhang , et al., “Model‐Based 3D Shape Reconstruction of Soft Robots via Distributed Strain Sensing,” Soft Robotics 12 (2025): 721–731, 10.1089/soro.2024.0158. 40406816

[5] X. Du , H. Cui , T. Xu , et al., “Reconfiguration, Camouflage, and Color‐Shifting for Bioinspired Adaptive Hydrogel‐Based Millirobots,” Advanced Functional Materials 30 (2020): 1909202, 10.1002/adfm.201909202.

[6] W. Haouas , M. Gauthier , and K. Rabenorosoa , “Miniaturized Soft Robotics: Recent Advances and Futures Opportunities,” Current Robotics Reports 5 (2024): 15–27, 10.1007/s43154-024-00109-3.

[7] P. Wang , X. Liu , and A. Song , “Actuation and Locomotion of Miniature Underwater Robots: A Survey,” Engineering 51 (2025): 195–214, 10.1016/j.eng.2024.10.022.

[8] H. Liu , Q. Guo , and W. Wang , “A Review of Magnetically Driven Swimming Microrobots: Material Selection, Structure Design, Control Method, and Applications,” Reviews on Advanced Materials Science 74 (2023): 101–130, 10.1515/rams-2023-0119.

[9] G. Kosa and P. Hunziker , “Small‐Scale Robots in Fluidic Media,” Advanced Intelligent Systems 1 (2019): 1900035, 10.1002/aisy.201900035.

[10] R. K. Katzschmann , J. DelPreto , R. MacCurdy , and D. Rus , “Exploration of Underwater Life With an Acoustically Controlled Soft Robotic Fish,” Science Robotics 3 (2018): aar3449, 10.1126/scirobotics.aar3449. 33141748

[11] G. Li , X. Chen , F. Zhou , et al., “Self‐Powered Soft Robot in the Mariana Trench,” Nature 591 (2021): 66–71, 10.1038/s41586-020-03153-z. 33658693

[12] A. Joshi , A. Kulkarni , and Y. Tadesse , “FludoJelly: Experimental Study on Jellyfish‐Like Soft Robot Enabled by Soft Pneumatic Composite (SPC),” Robotics 8 (2019): 56, 10.3390/robotics8030056.

[13] Y. Peng , H. Nabae , Y. Funabora , and K. Suzumori , “Controlling a Peristaltic Robot Inspired by Inchworms,” Biomimetic Intelligence and Robotics 4 (2024): 100146, 10.1016/j.birob.2024.100146.

[14] T. Cheng , G. Li , Y. Liang , et al., “Untethered Soft Robotic Jellyfish,” Smart Materials and Structures 28 (2019): 015019, 10.1088/1361-665X/aaed4f.

[15] H. Godaba , J. Li , Y. Wang , and J. Zhu , “A SOFT JELLYfish Robot Driven by a Dielectric Elastomer Actuator,” IEEE Robotics and Automation Letters 1 (2016): 624–631, 10.1109/LRA.2016.2522498.

[16] K. Marut , C. Stewart , T. Michael , A. Villanueva , and S. Priya , “A Jellyfish‐Inspired Jet Propulsion Robot Actuated By an Iris Mechanism,” Smart Materials and Structures 22 (2013): 094021, 10.1088/0964-1726/22/9/094021.

[17] J. Xiao , J. Duan , and J. Yu , “Design and Implementation of a Novel Biomimetic Robotic Jellyfish,” in Proceedings of the 2013 IEEE International Conference on Robotics and Biomimetics (ROBIO) IEEE, 2013, 988–993.

[18] Y. Almubarak , M. Punnoose , N. X. Maly , A. Hamidi , and Y. Tadesse , “KryptoJelly: A Jellyfish Robot With Confined, Adjustable Pre‐Stress, and Easily Replaceable Shape Memory Alloy NiTi Actuators,” Smart Materials and Structures 29 (2020): 075011, 10.1088/1361-665X/ab859d.

[19] Y. Zhou , H. Jin , C. Liu , E. Dong , M. Xu , and J. Yang , “A Novel Biomimetic Jellyfish Robot Based on a Soft and Smart Modular Structure (SMS),” in Proceedings of the 2016 IEEE International Conference on Robotics and Biomimetics (ROBIO) , (IEEE, 2016), 708–713.

[20] C. Yin , F. Wei , S. Fu , et al., “Visible Light‐Driven Jellyfish‐Like Miniature Swimming Soft Robot,” ACS Applied Materials & Interfaces 13 (2021): 47147–47154, 10.1021/acsami.1c13975. 34436851

[21] X. Lin and M. Han , “Recent progress in soft electronics and robotics based on magnetic nanomaterials,” Soft Science 3 (2023): 14, 10.20517/ss.2023.05.

[22] X. Yan , Q. Zhou , M. Vincent , et al., “Multifunctional Biohybrid Magnetite Microrobots for Imaging‐Guided Therapy,” Science Robotics 2 (2017): aaq1155, 10.1126/scirobotics.aaq1155. 33157904

[23] F. Wang , H. Zhang , and C. Liang , “Recent Progress and Perspective of Magnetic Miniature Soft Robot With Multimodal Locomotion,” Bioinspiration & Biomimetics 20 (2025): 053001, 10.1088/1748-3190/adfbcc. 40812360

[24] W. Hu , G. Z. Lum , M. Mastrangeli , and M. Sitti , “Small‐Scale Soft‐Bodied Robot With Multimodal Locomotion,” Nature 554 (2018): 81–85, 10.1038/nature25443. 29364873

[25] Y. Shao , A. Fahmy , M. Li , C. Li , W. Zhao , and J. Sienz , “Study on Magnetic Control Systems of Micro‐Robots,” Frontiers in Neuroscience 15 (2021): 736730, 10.3389/fnins.2021.736730. 34512256 PMC8432292

[26] M. Sokolich , D. Rivas , Y. Yang , M. Duey , and S. Das , “ModMag: A Modular Magnetic Micro‐Robotic Manipulation Device,” MethodsX 10 (2023): 102171, 10.1016/j.mex.2023.102171. 37122368 PMC10133746

[27] H. Liu , Q. Guo , W. Wang , et al., “A Review of Magnetically Driven Swimming Microrobots: Material Selection, Structure Design, Control Method, and Applications,” Reviews on Advanced Materials Science 62 (2023): 20230119, 10.1515/rams-2023-0119.

[28] H. C. M. Sun , P. Liao , T. Wei , L. Zhang , and D. Sun , “Magnetically Powered Biodegradable Microswimmers,” Micromachines 11 (2020): 404, 10.3390/mi11040404. 32294955 PMC7254493

[29] R. Pramanik , R. W. C. P. Verstappen , and P. R. Onck , “Nature‐Inspired Miniaturized Magnetic Soft Robotic Swimmers,” Applied Physics Reviews 11 (2024): 021312, 10.1063/5.0189185.

[30] G. Kósa , P. Jakab , G. Székely , and N. Hata , “MRI Driven Magnetic Microswimmers,” Biomedical Microdevices 14 (2012): 165–178, 10.1007/s10544-011-9594-7. 22037673 PMC3827905

[31] R. Tan , X. Yang , H. Lu , and Y. Shen , “One‐Step Formation of Polymorphous Sperm‐Like Microswimmers by Vortex Turbulence‐Assisted Microfluidics,” Nature Communications 15 (2024): 49043, 10.1038/s41467-024-49043-0. PMC11150408 38834563

[32] X. Ou , Y. Sheng , J. Huang , et al., “3D Printed Swordfish‐Like Wireless Millirobot,” Advanced Intelligent Systems 7 (2025): 200400206, 10.1002/aisy.202400206.

[33] C. Tian , L. Mao , P. Yang , H. Zhang , X. Meng , and H. Xie , “Carangiform‐Like Magnetic Milliswimmer With Negative Buoyancy for Agile 3D Navigation in Confined Fluid Environments,” Advanced Functional Materials 35 (2025): 2411017, 10.1002/adfm.202411017.

[34] Y. Wang , H. Chen , J. Law , X. Du , and J. Yu , “Ultrafast Miniature Robotic Swimmers With Upstream Motility,” Cyborg Bionic Systems 4 (2023): 0015, 10.34133/cbsystems.0015. 36939416 PMC10019906

[35] L. Zhang , J. J. Abbott , L. Dong , B. E. Kratochvil , D. Bell , and B. J. Nelson , “Artificial Bacterial Flagella: Fabrication and Magnetic Control,” Applied Physics Letters 94 (2009): 064107, 10.1063/1.3079655.

[36] X. Liu , L. Wang , Y. Xiang , et al., “Magnetic Soft Microfiberbots for Robotic Embolization,” Science Robotics 9 (2024): adh2479, 10.1126/scirobotics.adh2479. 38381840

[37] Y. Wang , D. Jin , and X. Liu , “Magnetic Microrobot With Drilling‐Sensing Dual Functionality for Targeted Biopsy of Deep‐Seated Tracheal Microlesions,” Advanced Materials 38 (2025): 14664, 10.1002/adma.202514664. 40984789

[38] X. Fan , Q. Chen , M. Li , et al., “Machining Swarf Formation–Inspired Fabrication of Ferrofluidic Helical Miniature Robots With Multimodal Locomotion Capability,” Science Advances 11 (2025): ads4411, 10.1126/sciadv.ads4411. PMC12227062 40614200

[39] Z. Ren , W. Hu , X. Dong , and M. Sitti , “Multi‐Functional Soft‐Bodied Jellyfish‐Like Swimming,” Nature Communications 10 (2019): 2703, 10.1038/s41467-019-10549-7. PMC6606650 31266939

[40] S. Wu , Q. Ze , R. Zhang , et al., “Symmetry‐Breaking Actuation Mechanism for Soft Robotics and Active Metamaterials,” ACS Applied Materials & Interfaces 11 (2019): 41649–41658, 10.1021/acsami.9b13840. 31578851

[41] J. Ye , Y.‐C. Yao , J.‐Y. Gao , et al., “LM‐Jelly: Liquid Metal Enabled Biomimetic Robotic Jellyfish,” Soft Robotics 9 (2022): 1098–1107, 10.1089/soro.2021.0132. 35486839

[42] G. Mao , D. Schiller , D. Danninger , et al., “Ultrafast Small‐Scale Soft Electromagnetic Robots,” Nature Communications 13 (2022): 4456, 10.1038/s41467-022-32123-4. PMC9363453 35945209

[43] B. H. Kim , K. Li , J.‐T. Kim , et al., “Three‐Dimensional Electronic Microfliers Inspired by Wind‐Dispersed Seeds,” Nature 597 (2021): 503–510, 10.1038/s41586-021-03847-y. 34552257

[44] Q. Le , J. Bu , Y. Qu , B. Zhu , and T. Du , “Computational Biomimetics of Winged Seeds,” ACM Transactions on Graphics 43 (2024): 1–13, 10.1145/3687899.

[45] J. H. Costello , S. P. Colin , J. O. Dabiri , B. J. Gemmell , K. N. Lucas , and K. R. Sutherland , “The Hydrodynamics of Jellyfish Swimming,” Annual Review of Marine Science 13 (2021): 375–396, 10.1146/annurev-marine-031120-091442. 32600216

[46] B. Sun , R. Jia , H. Yang , et al., “Magnetic Arthropod Millirobots Fabricated by 3D‐Printed Hydrogels,” Advanced Intelligent Systems 4 (2022): 2100139, 10.1002/aisy.202100139.

[47] G. E. Budd and M. J. Telford , “The Origin and Evolution of Arthropods,” Nature 457 (2009): 812–817, 10.1038/nature07890. 19212398

[48] R. Liang , G. Xu , Z. Teng , et al., “A General Arthropod Joint Model and its Applications in Modeling Human Robotic Joints,” IEEE Access 9 (2021): 7814–7822, 10.1109/ACCESS.2021.3049469.

[49] A. W. Mahoney , N. D. Nelson , K. E. Peyer , B. J. Nelson , and J. J. Abbott , “Behavior of Rotating Magnetic Microrobots Above the Step‐Out Frequency With Application to Control of Multi‐Microrobot Systems,” Applied Physics Letters 104 (2014): 144101, 10.1063/1.4870933.

[50] J. Giltinan , P. Katsamba , W. Wang , E. Lauga , and M. Sitti , “Selectively Controlled Magnetic Microrobots With Opposing Helices,” Applied Physics Letters 116 (2020): 134101, 10.1063/1.5143007.

