# Supplementary material for: A Magnetic‐Driven Self‐Rotation‐Stabilized Miniature Swimming Robot
Source: Adv Sci (Weinh). 2026 Sep 9:e77382. Online ahead of print. doi: 10.1002/advs.77382 (PMC13555360; doi:10.1002/advs.77382)
Supplement: Supplementary file 1 [file ADVS-9999-e77382-s005.docx]

Supplementary Information for

A Magnetic-driven Self-rotation-stabilized Miniature Swimming Robot

Fujun Wang,^1^ Chuhan Zhang,^1^ Hao Zhang,^1^ Yanling Tian,^2^ Cunman Liang ^1^*

^1^ Key Laboratory of Mechanism Theory and Equipment Design of Ministry of Education, School of Mechanical Engineering, Tianjin University, Tianjin 300354, People’s Republic of China.

^2^ School of Engineering, University of Warwick, Coventry CV4 7AL, UK.

*Corresponding author. Email: lcm@tju.edu.cn

**This PDF file includes:**

Notes S1 to S4

Figures. S1 to S29

Table S1

Reference [1] to [4]

**Other Supplementary Materials for this manuscript include the following:**

Movies S1 to S6

**Supplementary Text**

**Supplementary Note 1. Theoretical model of swimming locomotion**

Based on the study of jellyfish dynamics by McHenry et al. [1], we established the swimming model of the robot. The force analysis of the robot during the swimming process is shown in Figure S24. The forces acting on the robot in the swimming state include the thrust *F_z_* generated by the paddling motion, the fluid drag *D_z_*, the added mass force *A_z_*, the buoyant force *F_b_*, and the gravitational force *G*. When the robot's four magnetic legs perform a paddling motion to strike the fluid medium, they experience a reaction force *F_p_* from the fluid. The component of the reaction force *F_p_* in the direction of the robot's motion, denoted as *F_z_*, is responsible for driving the robot's swimming. The relationship between the thrust *F_z_* and the reaction force *F_p_* can be expressed as:

In the equation, *θ* denotes the instantaneous swing angle of the magnetic leg during the robot’s motion, and *φ* is the inclination angle induced by the leg’s oblique magnetization direction. Figure S25 shows the simplified model of the robot in the bell-shaped posture; the thrust *F_z_* acting on the robot can be expressed as:

In the equation, *C_l_* is the drag coefficient for the robot striking the fluid medium, with a value of 1.98 [2]. *ρ_L_* is the density of the fluid medium, and d*S* is the area of a micro-ring on the robot. The area of the micro-ring d*S* can be expressed as:

$$dS=2\pi rdr$$

In the equations, d*r* is the width of the micro-ring. Thus, the thrust *F_z_* generated by the robot can be expressed as:

where *R* is the size radius of the robot. The fluid drag *D_z_* acting on the robot can be expressed as:

$$D_{z}=\frac{1}{2}C_{d}S_{p}\rho_{L}v^{2}$$

where *v* is the robot's swimming velocity, and *C_d_* is the drag coefficient of the fluid [3], which can be expressed as:

$$C_{d}=\frac{24}{\mathrm{Re}_{B}}\left(1+0.173\mathrm{Re}_{B}^{0.657}\right)+\frac{0.413}{1+163000\mathrm{Re}_{B}^{-1.09}}$$

where Re*_B_* is the Reynolds number of the robot in the fluid environment, which can be expressed as:

$$\mathrm{Re}_{B}=\frac{\rho_{L}vl_{r}}{\mu}$$

where *l_r_* is the robot's characteristic length, and *μ* is the dynamic viscosity of the fluid medium. *S_p_* is the projected area of the robot in the swimming direction, which can be expressed as:

$$S_{p}=\pi\left[\left(L_{l}+L_{j}\right)\sin\theta\right]^{2}$$

The added mass force *A_z_* is the additional force generated when the robot accelerates the surrounding fluid medium as it swims. The calculation formula for *A_z_* is:

$$A_{z}=\delta\rho V_{c}\frac{dv}{dt}$$

where *δ* is the added mass coefficient, reflecting the effect of the robot's shape on the surrounding fluid, and can be expressed as:

$$\delta=\tan^{-1.4}\theta$$

*V_c_* is the volume of the bell-shaped cavity when the robot's envelope deforms into a bell shape, which is related to the deformation angle of the robot's magnetic legs, and can be expressed as:

In addition, the robot experiences buoyant force *F_b_* generated by the fluid medium, which can be expressed as:

$$F_{b}=\rho_{L}gV_{r}$$

where *g*=9.8 m/s^2^ and *V_r_* is the robot's volume. The swimming process of robot can be divided into four phases:

**(1) Phase I: Propulsion phase**
In this phase, the robot is subject to the thrust *F_z_*, fluid drag *D_z_*, added mass force *A_z_*, buoyant force *F_b_*, and gravity *G.* The robot's swimming dynamics equation can be expressed as:

$$F_{z}-D_{z}-A_{z}-G+F_{b}=m\frac{dv}{dt}$$

where *m* is the robot's mass.

**(2) Phase II: Bell-shaped posture gliding phase**

When the robot reaches its maximum contracted state, the four magnetic legs stop striking the fluid medium, and the robot maintains the bell-shaped posture while gliding for a period of time. At this point, the thrust *F_z_* is 0, and the robot's swimming dynamics equation can be expressed as:

$$F_{b}-D_{z}-A_{z}-G=m\frac{dv}{dt}$$

**(3) Phase III: Extension phase**

As the robot gradually extends from the bell-shaped state to the planar state, the four magnetic legs strike the fluid medium in the opposite direction. At this point, the thrust *F_z_* is opposite to the swimming direction, and the robot's swimming dynamics equation can be expressed as:

$$-F_{z}-D_{z}-A_{z}-G+F_{b}=m\frac{dv}{dt}$$

**(4) Phase IV: Planar posture gliding phase**

When the robot reaches its maximum extended state, i.e., the planar posture, the four magnetic legs stop striking the fluid medium and the robot maintains the planar posture while gliding. At this point, the thrust *F_z_* is 0, and the robot no longer encloses the fluid medium in the planar posture, meaning the added mass force *A_z_* is also 0. Therefore, the robot's swimming dynamics equation can be expressed as:

$$F_{b}-D_{z}-G=m\frac{dv}{dt}$$

Based on the established swimming motion model of the robot, the following conclusions can be drawn: The key factors influencing the robot's swimming speed are the thrust *F_z_* and fluid drag *D_z_*. Increasing the thrust *F_z_*, such as by increasing the speed at which the robot strikes the fluid medium during the paddling motion, or by increasing the amplitude of the robot's paddling motion (i.e., increasing the bending angle of the magnetic legs under the magnetic field), can enhance the robot's swimming speed.

**Supplementary Note 2. Theoretical model of self-rotation motion**

To establish the relationship between the self-rotation angular velocity of the self-rotating swimming soft robot and the frequency of the external magnetic field, a self-rotation motion model is developed in this section. The relationship between the robot's self-rotation and propulsive force is then analyzed. The circumferential component *F_c_* of the paddling drag force *F_p_* generates a rotational moment *M_c_* that drives the robot to self-rotate. The relationship between *F_c_* and *F_p_* can be expressed as

(S-17)

Because the soft film between adjacent magnetic legs is much thinner than the legs themselves, its contribution to the circumferential force *F_c_* is negligible. Therefore, the film is neglected in the self-rotation model, and only the magnetic legs are considered. Since the four magnetic legs are subjected to identical loading conditions, the force analysis is performed for one representative leg, as shown in Figure S26.

The circumferential force *F_c_* is given by

(S-18)

where *S_L_* is the trapezoidal surface area of one magnetic leg during paddling. Based on the hydrodynamic analysis reported by Hoerner et al. [2], *C_l_* is taken as 1.98. The quantity *u_c_* denotes the instantaneous linear velocity at the center of the magnetic leg.

The relationship between the rotational moment *M_c_* and the circumferential force *F_c_* is

$M_{c}=F_{c}r_{c}$ (S-19)

where *r_c_* is the distance from the center of the magnetic leg to the center of the robot.

The circumferential hydrodynamic drag *D_c_* acting on one magnetic leg can be expressed as

$D_{z}=\frac{1}{2}C_{d}S_{Lp}\rho\omega_{z}^{2}r_{c}^{2}$ (S-20)

where *ω_z_* is the self-rotation angular velocity of the robot.

The projected area *S_Lp_* of one magnetic leg in the circumferential direction is

(S-21)

During self-rotation, the robot is assumed not to entrain the surrounding fluid as a rigidly rotating fluid mass; therefore, added-mass effects are neglected. Accordingly, the circumferential dynamics are governed only by the circumferential force *F_c_* generated as the paddling legs strike the fluid and the opposing circumferential hydrodynamic drag *D_c_*.

Similarly, one self-rotating swimming cycle can be divided into four phases:

**(1) Phase I: Propulsion**

During this phase, the robot is simultaneously subjected to the circumferential force *F_c_* and the circumferential hydrodynamic drag *D_c_*. Because *F_c_* exceeds *D_c_*, the robot accelerates under the resulting rotational moment. The equation governing self-rotation in this phase is

$4F_{c}r_{c}-4D_{c}r_{c}=I_{3}\frac{d\omega}{dt}$ (S-22)

where *I_z_* is the robot's moment of inertia about its axial direction.

**(2) Phase II: Gliding in the bell-shaped configuration**

When the robot reaches its maximum contracted state, the four magnetic legs cease striking the fluid, and the robot glides in the bell-shaped configuration for a finite duration. At this stage, *F_c_* is zero, and only the resistive moment associated with *D_c_* acts on the robot; consequently, its angular velocity decreases. The corresponding equation of self-rotation is

$-4D_{c}r_{c}=I_{3}\frac{d\omega}{dt}$ (S-23)

**(3) Phase III: Extension**

As the robot extends from the bell-shaped configuration to the planar configuration, the four magnetic legs strike the fluid in the opposite direction. The moment generated by *F_c_* is therefore opposite to the self-rotation direction, producing a braking effect and further reducing the angular velocity. The governing equation in this phase is

$-4F_{c}r_{c}-4D_{c}r_{c}=I_{3}\frac{d\omega}{dt}$ (S-24)

**(4) Phase IV: Gliding in the planar configuration**

When the robot reaches its maximum extension, i.e., the planar configuration, the four magnetic legs cease striking the fluid, and the robot glides. The corresponding equation of self-rotation is

$I_{3}\frac{d\omega}{dt}=0$ (S-25)

Several conclusions can be drawn from the self-rotation dynamic model. The robot's rotational motion is governed by the circumferential force *F_c_* and the circumferential hydrodynamic drag *D_c_*. Increasing either the velocity at which the paddling legs strike the fluid during propulsion or the paddling amplitude increases the robot's self-rotation angular velocity.

**Supplementary Note 3. Dynamic response model of robot joints**

The locomotion performance of the robot is closely related to the dynamic characteristics of its flexible joints. The dynamic response speed of these joints to an external magnetic field directly determines the maximum driving frequency of the magnetic field, thereby indirectly influencing the robot’s overall locomotion performance. In this work, based on the Lagrangian equation, a dynamic response model of the flexible joints of MSMSR is established.

The ratio of the trapezoidal magnetic leg length *L_l_* to the flexible joint length *L_j_* is less than 10, indicating that it belongs to a slender-rod-type flexible element. Under the action of a uniform external magnetic field, the magnetic leg of the robot is subjected to a bending moment. In this case, the flexible joint can be regarded as a cantilever beam with one end fixed and the other end having both its angle and position varying with the rotation angle. For such a cantilever beam, the instantaneous center of motion of the endpoint can be readily determined. Based on the concept of the instantaneous center, the beam model of the robot’s flexible joint is established, as illustrated in Figure S27 One end of the flexible joint AD is fixed at the robot’s center, while the other end D rotates about the instantaneous center of velocity O.

After deformation of the flexible joint, the tangential displacement w of the free end point D is given by

$w=\frac{F_{1}L_{j}^{3}}{3EI}-\frac{ML_{j}^{2}}{2EI}$ (S-26)

The circumferential displacement e is given by

$e=\frac{8F_{1}^{2}L_{j}^{5}-25MF_{1}L_{j}^{4}+20M^{2}L_{j}^{3}}{120E^{2}I^{2}}$ (S-27)

where *F*_1_ is the tangential force acting on the flexible joint, *M* is the bending moment applied to the flexible joint, *E* is the Young’s modulus of the flexible joint, and *I* is its area moment of inertia.

The rotation angle *α_j_* of the free end D about the instantaneous center O is given by

$n=\frac{L_{OD}}{L_{j}}$ (S-28)

where *L*_OD_ is the distance from the instantaneous center O to the free end D.

The relationship between the lateral displacement *w* and *α_j_* is given by

$\alpha_{j}L_{OD}=w$ (S-29)

From Eq. (S-26)–(S-29), *α_j_* and *M* can be solved as

$\alpha_{j}=\frac{F_{1}L_{j}^{2}}{6EI(2n-1)}$ (S-30)

$M=\frac{F_{1}L_{j}(2-3n)}{3(1-2n)}$ (S-31)

According to the geometric relationship shown in Figure S25, the location of the equivalent hinge O′ in the flexible joint beam model must satisfy

$\tan\frac{\theta}{2}=\frac{e}{w}$ (S-32)

$\gamma L_{j}\sin(\frac{\theta}{2})=\frac{\sqrt{w^{2}+e^{2}}}{2}$ (S-33)

where *θ* is the rotation angle of point D about the equivalent hinge O′, and *γ* is the characteristic radius coefficient of the flexible joint beam model. The distance from point D to the equivalent hinge O′ is expressed as *γL_j_*. The characteristic radius coefficient *γ* of the flexible joint beam model is obtained as

$\gamma=\frac{15n^{2}}{18n^{2}-3n+2}$ (S-34)

Since the flexible joint of the robot can be modeled as a cantilever beam with a torque applied at the free end, we take *γ* = 0.7346. By modeling the flexible joint as an equivalent hinge with a torsional spring, the equivalent torsional stiffness coefficient is obtained as

$K_{d}=\frac{4EI\gamma^{2}(3n^{2}-3n)+1}{n^{2}L_{j}}=1.5164\frac{EI}{L_{j}}$ (S-35)

Under pure bending moment, the elastic potential energy stored in the elastic deformation of the flexible joint beam is equal to the work done by the bending moment *M*.

$U_{j}=\int_{0}^{\alpha} M_{M}d\alpha=\frac{1}{2}K_{d}\theta^{2}=0.57573\frac{EI}{L_{j}}\theta^{2}=0.057573\frac{EI}{L_{j}}\theta^{2}$ (S-36)

The kinetic energy of the flexible joint and magnetic leg consists of two parts: one is the kinetic energy of the flexible joint, the other is the kinetic energy of the magnetic leg. When the flexible joint rotates about the hinge, its rotational inertia *J*_1_ is given by

$J_{1}=m_{j}r_{j}^{2}=(\frac{1}{2}-\gamma)^{2}L_{j}^{3}\rho_{j}W_{j}H_{j}$ (S-37)

where *m_j_* is the mass of the flexible joint, *r_j_* is the distance from the free end of the flexible joint to the equivalent hinge O′, *ρ_j_* is the density of the flexible joint, *W_j_* is its width, and *H_j_* is its thickness.

The rotational inertia *J*_2_ of the magnetic leg about the equivalent hinge O′ is given by

(S-38

where *ρ_l_* is the density of the magnetic leg, *L_c_* is the distance from the center of mass of the magnetic leg to the top base of the trapezoidal magnetic leg, *H_l_* is the thickness, and *W_l_*_1_ and *W_l_*_2_ are the widths of the top and bottom bases of the trapezoidal magnetic leg, respectively. In addition, the magnetic leg also rotates about its own center of mass, and its rotational inertia *J*_3_ is given by

(S-39)

Thus, the total kinetic energy of the flexible joint beam model is

$T=\frac{1}{2}J\theta'^{2}=\frac{1}{2}(J_{1}+J_{2}+J_{3})\theta'^{2}$ (S-40)

According to the Lagrangian equation, the dynamic equation of the flexible joint beam model can be expressed as

$\frac{d}{dt}\left(\frac{\partial L}{\partial\theta'}\right)-\frac{\partial L}{\partial\theta}=(J_{1}+J_{2}+J_{3})\theta''+0.057573K_{d}\theta=0$ (S-41)

The first-order natural frequency of the flexible joint cantilever beam model is obtained as

$f=\frac{1}{2\pi}\sqrt{\frac{K_{d}}{J_{1}+J_{2}+J_{3}}}$ (S-42)

**Supplementary Note 4. Theoretical model for self-rotation stability**

For analytical convenience, the self-rotation of the swimming soft robot is simplified as the rotation of a rigid body subjected to hydrodynamic effects (Figure S28). The angular-velocity vector of the robot can therefore be written as

$\omega_{x}=\omega_{x}e_{x}+\omega_{y}e_{y}+\omega_{z}e_{z}$ (S-43)

where ***e_x_***, ***e_y_***, and ***e_z_*** are the unit vectors along the X, Y, and Z directions, respectively, and *ω_x_*, *ω_y_*, and *ω_z_* are the corresponding components of the robot's angular velocity.

The robot generally operates in a flowing-water environment and may therefore be subjected to transverse-flow impacts. Whether it can recover a stable state after such a disturbance is a critical requirement for reliable operation [4]. Accordingly, a theoretical motion-stability model is established for the self-rotating swimming soft robot.

As shown in Figure S29, a transverse-flow impact during self-rotating swimming induces perturbations about both the X and Y axes. According to Euler's equations of rotation, the angular-velocity vector can be expressed as

$\omega=\Lambda'_{x}e_{x}+\Lambda'_{y}e_{y}+\omega_{z}e_{z}$ (S-44)

where Λ*_x_* and Λ*_y_* are the perturbation angles about the X and Y axes.

Euler's equations of robot can be written as

$\left\{\begin{matrix} T_{x}=I_{x}\Lambda''_{x}-(I_{y}-I_{z})\Lambda'_{y}\omega_{z} \\ T_{y}=I_{y}\Lambda''_{y}-(I_{z}-I_{x})\omega_{z}\Lambda_{x} \\ T_{z}=I_{z}\omega'_{z}-(I_{x}-I_{y})\Lambda'_{x}\Lambda_{y} \end{matrix}\right.$ (S-45)

Accordingly, when the robot is perturbed, the torque acting on it can be written as

$\mathbf{T}=\mathbf{T}(\Lambda,\Lambda')$ (S-46)

Based on the derivation presented in Ref. [4], we yield the governing equations for robot stability:

$\left(\begin{matrix} \alpha''_{x} \\ \alpha''_{y} \end{matrix}\right)+\left(\begin{matrix} \beta_{0}-\omega_{0}i & 0 \\ 0 & \beta_{0}+\omega_{0}i \end{matrix}\right)\left(\begin{matrix} \alpha'_{x} \\ \alpha'_{y} \end{matrix}\right)+\left(\begin{matrix} \varepsilon& 0 \\ 0 & \varepsilon\end{matrix}\right)\left(\begin{matrix} \alpha_{x} \\ \alpha_{y} \end{matrix}\right)=0$ (S-47)

where *α_x_* and *α_y_* are the disturbance angle after decoupling, *β*_0_=*πμr*^2^/8*I_x_*Re*_B_*, *ω*_0_=(*I_z_*-*I_y_*)/*I_x_ω_T_*, *ε*=*Wd*/*I_x_*, *μ* is the dynamic viscosity of water, *r* is the radius of robot, *ω_T_* is the rotational angular velocity of the robot's rotation along the *Z* direction, Re*_B_* is the Reynolds number in water, *W* is the work done by the magnetic force on the robot, *I_x,y,z_* are the moment of inertias for directions X, Y and Z, and *d* is the distance between the center of gravity and the center of pressure. The resulting system consists of two decoupled second-order damped oscillation equations. Solving the associated characteristic equations gives the four eigenvalues. And for subsequent solution and analysis, the eigenvalue expressions are normalized as

$\lambda_{1,2}=-\frac{\beta_{0}}{2}(1-\frac{\omega_{0}}{\beta_{0}}i)\pm\sqrt{\left(1-\frac{\omega_{0}}{\beta_{0}}i\right)^{2}-\frac{4\varepsilon}{\beta_{0}^{2}}}$ (S-48)

$\lambda_{3,4}=-\frac{\beta_{0}}{2}(1+\frac{\omega_{0}}{\beta_{0}}i)\pm\sqrt{\left(1+\frac{\omega_{0}}{\beta_{0}}i\right)^{2}-\frac{4\varepsilon}{\beta_{0}^{2}}}$ (S-49)

For the robot to recover its stable state after an external flow impact, the perturbation angles must decay with time and eventually approach zero. According to the stability criterion for linear time-invariant systems, all four eigenvalues must have negative real parts. The stability factor *Ω* is therefore defined as

$\Omega=\min\left[-\frac{2Real(\lambda_{1,2,3,4})}{\beta_{0}}\right]=\min\left[Real\left((1\pm\frac{\omega_{0}}{\beta_{0}}i)\pm\sqrt{\left(1\pm\frac{\omega_{0}}{\beta_{0}}i\right)^{2}-\frac{4\gamma}{\beta_{0}^{2}}}\right)\right]$ (S-50)

where Real(·) denotes the real part of a complex number. The criterion for recovery to a stable state is therefore *Ω* > 0.

Solving the differential equations gives the time-dependent perturbation angle as

$\Lambda(t)=\sum_{i=1}^{4} C_{i}e^{\lambda_{i}t}$ (S-51)

where *C_i_* is a constant, and *λ_i_* is the eigenvalue corresponding to the robot's different swimming speeds and rotational angular velocities. When the robot is subjected to a transverse-flow impact, variations in its swimming speed and self-rotation angular velocity lead to different eigenvalues. The theoretical analysis therefore indicates that both the maximum perturbation angle and the settling time required to recover stability depend on the robot's swimming speed and self-rotation angular velocity.

***Figure S1.* Fabrication process of MSMSR.**

***Figure S2.* Swimming performance of MSMSR predicted by theoretical model under a magnetic field of 17.8 Hz (top) and 18.5 Hz (bottom).** (A) multi-cycle instantaneous velocity and displacement variation curves. (B) single-step swimming performance curves.

***Figure S3.* Locomotion process of MSMSR under magnetic fields of different frequencies.** (A) Medium frequency drive magnetic field: Δ*t*_2_=Δ*t*_4_=0, MSMSR only has two phases, Phase 1 (contraction) and Phase 3 (extension), during which it reaches the maximum speed. (B) High frequency drive magnetic field: Δ*t*_2_=Δ*t*_4_<0, Δ*t*_1_*^'^*=Δ*t*_3_*^'^*<Δ*t*_1_=Δ*t*_3_, *θ*_0_*^'^*<*θ*_0_. The durations of Phase 1 and Phase 3 gradually shorten, and the robot's magnetic legs cannot fully extend to the planar posture within one swimming cycle. Thus, the swimming speed decreases as the magnetic field frequency increases.

***Figure S4.* Tensile testing of materials.** (A) Dumbbell-shaped specimen. (B) Tensile test procedure.

***Figure S5.* Results of tensile test.**

***Figure S6*. Natural frequency testing experiment of MSMSR.** (A) schematic image. (B) physical image.

***Figure S7.* Locomotion performance experiment of MSMSR (10 mT, 17.8 Hz).** (A) Locomotion process. (B) Instantaneous velocity and displacement during a single locomotion cycle.

***Figure S8.* Locomotion performance experiment of MSMSR (10 mT, 18.5 Hz).** (A) Locomotion process. (B) Instantaneous velocity and displacement during a single locomotion cycle.

***Figure S9*. Physical prototypes of robots with different magnetization angles (from left to right: MSMSR-1, MSMSR, and MSMSR-2, corresponding to 15°, 30°, and 45°, respectively).**

***Figure S10*. Locomotion process of MSMSR-1 under different magnetic field driving frequencies.** (A) 13 Hz. (B) 17 Hz.

***Figure S11*. Locomotion process of MSMSR-2 under different magnetic field driving frequencies.** (A) 13 Hz. (B) 17 Hz.

***Figure S12*. Relationship between the driving magnetic field frequency and robots’ self-rotational angular velocity.** (A) MSMSR-1. (B) MSMSR-2.

***Figure S13.* Comparison of the precise directional swimming angle ranges of MSMSR and Non-self-rotating MSR.** (A) MSMSR. (B) Non-self-rotating MSR.

***Figure S14.* Swimming angle of MSMSR under different vertical magnetic field frequencies.**

***Figure S15.* Correlation between the MSMSR's stabilization time and its rotational angular velocity through theoretical prediction.**

***Figure S16.* Relationship between the stability parameters with MSMSR locomotion parameters (self-rotational angular velocity and swimming speed).** (A) Relationship between the parameter *ω*_0_/*β*_0_ with self-rotational angular velocity, (B) Relationship between the parameter 4*ε*/*β*_0_^2^ with swimming speed.

***Figure S17.* Disturbance angle and stabilization time of MSMSR-1 under different lateral water flow impact.** (A) Disturbance angle. (B) Stabilization time.

***Figure S18.* Disturbance angle and stabilization time of MSMSR-2 under different lateral water flow impact.** (A) Disturbance angle. (B) Stabilization time.

***Figure S19.* Schematic of the water flow impact setup under different inclination angles.** (A) downward inclination of 15°. (B) upward inclination of 15°.

***Figure S20.* Experimental results of the MSRs under upward 15° inclined water flow impacts (120 mm/s) at different magnetic field driving frequencies.** (A) Disturbance Angle. (B) Stabilization time.

***Figure S21.* Experimental results of the MSRs under downward 15° inclined water flow impacts (120 mm/s) at different magnetic field driving frequencies.** (A) Disturbance Angle. (B) Stabilization time.

***Figure S22.* Process of the proposed MSMSR swimming to the target area and monitoring water temperature variations.** (A) Experimental procedure. (B) Changes in the sensor before and after the experiment.

***Figure S23.* Magnetic driving system.**

***Figure S24.*** **Forces analysis of robot swimming mode.**

***Figure S25.*** **Simplified model of the robot bell-shaped attitude.** (A) Thrust *F_z_* is solved by integration. (B) Simplified model geometrical parameters.

***Figure S26.* Circumferential force analysis on a single leg of a robot in autorotation motion.**

***Figure S27.* Analytical modeling of flexible joint beam.**

***Figure S28.* Perturbation model of a robot.**

***Figure S29.* Force analysis during robot perturbation.**

***Table S1*. The main performance comparison of relative MSRs and our robot**

| **Actuator** | **Wireless**  **controllability** | **Swimming form** | **Body length**  **(BL, mm)** | **Speed (BL/s)** | **Ability to resist undercurrents** | **Self-rotating** | **Manufacturing difficulty** | **Ref** |
| --- | --- | --- | --- | --- | --- | --- | --- | --- |
| Pneumatic (Air) | No | Pulse propulsion | 220 | 0.72 | No | No | Hard | (11) |
| Dielectric elastomers | No | Pulse propulsion | 156 | 0.06 | No | No | Easy | (13) |
| Dielectric elastomers | No | Pulse propulsion | 270 | — | No | No | Middle | (14) |
| Motor | No | Pulse propulsion | 80 | 3.8 | No | No | Hard | (15) |
| SMA wire | No | Pulse propulsion | 210 | 0.28 | No | No | Hard | (17) |
| SMA wire | No | Pulse propulsion | 216 | 0.20 | No | No | Middle | (18) |
| Light | Yes | Pulse propulsion | 10 | 0.34 | No | No | Easy | (19) |
| Magnetic | Yes | Swing | 0.1 | 0.23 | No | No | Hard | (27) |
| Magnetic | Yes | Swing | 20 | 0.51 | No | No | Hard | (28) |
| Magnetic | Yes | Spiral | 0.05 | 0.06 | No | Yes | Hard | (30) |
| Magnetic | Yes | Spiral | 6 | 0.75 | No | Yes | Easy | (31) |
| Magnetic | Yes | Pulse propulsion | 5 | 10 | No | No | Hard | (35) |
| Magnetic | Yes | Pulse propulsion | 28 | 0.47 | No | No | Easy | (36) |
| Magnetic | Yes | Pulse propulsion | 30 | 0.2 | No | No | Middle | (37) |
| Magnetic | Yes | Pulse propulsion | 20 | 1.25 | No | No | Middle | (38) |
| Magnetic | Yes | Pulse propulsion | 15 | **2.92** | Yes | **Yes** | **Easy** | **This work** |

**References**

1. M. J. McHenry and J. Jed, “The ontogenetic scaling of hydrodynamics and swimming performance in jellyfish (*Aurelia aurita*),” *Journal of Experimental Biology* 206 (2003): 4125-4137, https://doi.org/10.1242/jeb.00649.
2. S. F. Hoerner, *Fluid-Dynamic Drag: Practical Information on Aerodynamic Drag and Hydrodynamic Resistance* (Hoerner Fluid Dynamics, 1965), 455.
3. R. Turton and O. Levenspiel, “A short note on the drag correlation for spheres,” *Powder Technology* 47 (1986): 83–86, https://doi.org/10.1016/0032-5910(86)80012-2.
4. B. H. Kim, K. Li, J.-T. Kim, et al., “Three-dimensional electronic microfliers inspired by wind-dispersed seeds,” *Nature* 597 (2021): 503–510, https://doi.org/10.1038/s41586-021-03847-y.
